# Global Scale Transcriptional Profiling of Two Contrasting Barley Genotypes Exposed to Moderate Drought Conditions: Contribution of Leaves and Crowns to Water Shortage Coping Strategies

**DOI:** 10.3389/fpls.2016.01958

**Published:** 2016-12-27

**Authors:** Pavel Svoboda, Anna Janská, Vojtěch Spiwok, Ilja T. Prášil, Klára Kosová, Pavel Vítámvás, Jaroslava Ovesná

**Affiliations:** ^1^Division of Crop Genetics and Breeding, Crop Research InstitutePrague, Czechia; ^2^Faculty of Science, Charles University in PraguePrague, Czechia; ^3^Faculty of Food and Biochemical Technology, University of Chemistry and TechnologyPrague, Czechia

**Keywords:** barley, drought, microarray, leaf, crown, Tadmor, Amulet

## Abstract

Drought is a serious threat for sustainable agriculture. Barley represents a species well adapted to environmental stresses including drought. To elucidate the adaptive mechanism of barley on transcriptional level we evaluated transcriptomic changes of two contrasting barley cultivars upon drought using the microarray technique on the level of leaves and crowns. Using bioinformatic tools, differentially expressed genes in treated vs. non-treated plants were identified. Both genotypes revealed tissue dehydration under drought conditions as shown at water saturation deficit and osmotic potential data; however, dehydration was more severe in Amulet than in drought-resistant Tadmor under the same ambient conditions. Performed analysis showed that Amulet enhanced expression of genes related to active plant growth and development, while Tadmor regarding the stimulated genes revealed conservative, water saving strategy. Common reactions of both genotypes and tissues included an induction of genes encoding several stress-responsive signaling proteins, transcription factors as well as effector genes encoding proteins directly involved in stress acclimation. In leaf, tolerant cultivar effectively stimulated mainly the expression of genes encoding proteins and enzymes involved in protein folding, sulfur metabolism, ROS detoxification or lipid biosynthesis and transport. The crown specific reaction of tolerant cultivar was an enhanced expression of genes encoding proteins and enzymes involved in cell wall lignification, ABRE-dependent abscisic acid (ABA) signaling, nucleosome remodeling, along with genes for numerous jasmonate induced proteins.

## Introduction

Drought, a primary threat for sustainable agriculture, is responsible for the largest loses of plant production at a global scale (Boyer, 1982; Araus et al., 2008). In addition, considering projected climatic changes, we can presume that impact of this stressor on agricultural production will escalate in the future (Beddington et al., 2011; Field, 2012). In negative contrast with this phenomenon, there is a rapid growth of human population, which is anticipated to overcome nine billion people in the horizon of 40 years (Bruinsma, 2009; Godfray et al., 2010; Gregory and George, 2011). To ensure food security during comming years, it will be necessary to breed crop cultivars capable to withstand longer periods of water withholding while maintaining good yields in such adverse conditions.

Tolerance relies on the inherent ability of the plant to sustain growth (likely at a reduced rate) even when the conditions are unfavorable for the maintenance of basic plant processes (Peleg et al., 2011). Unfortunately, drought tolerance is a very complex trait including series of physiological and biochemical responses (Cattivelli et al., 2008). To be more complicated, breeding efforts are targeted to gain good yield potential under water scarcity instead of simple surviving. Therefore, creating tolerant genotypes is great challenge for genetic engineering (Cattivelli et al., 2008).

Great efforts were recently dedicated to elucidation of mechanisms involved in drought tolerance. Genes coded for proteins related to osmoregulation (Δ1-pyrroline-5-carboxylate synthetase, glycine betaine), mitigating oxidative damage (superoxide dismutase, glutathione *S*-transferase), ionic balance (AtHKT1, AtNHX1 transporters) or genes coded for proteins with regulatory and signaling function (DREB, NAC transcriptional factors) were described (Peleg et al., 2011) and succesfully used to conferring drought tolerance by their transferring into different plant species (Pellegrineschi et al., 2002, 2004; Umezawa et al., 2006). However, despite this progress, there is still the limited knowledge about mechanisms that would allow crop plants to achieve good yield potential under water shortage.

Barley is one of the model organisms for genetic and physiological studies, exhibiting by dint of genotypes variability a wide flexibility in adaptation to environmental conditions (Nevo, 1992). Considering the harvesting area, it is the fourth most planted crop worldwide (Faostat, 2016), with a great impact for livestock production, brewing and food industries. It is cultivated in many developing countries, where it is often exposed to extreme drought, which significantly affects its production (Ceccarelli, 1994). Elucidation of mechanisms which facilitate barley to survive under dehydration stress could lead to better understanding of genetic bases linked to plant adaptability to drought in general, and, therefore, enables more efficient manners of breeding methods utilization in drought tolerance improvement. One possible way to achieve such goal is genome-scale expression profiling via microarray technology.

DNA microarrays found a broad range of applications in monitoring of expression changes in plants after exposure to various stresses (Kawasaki et al., 2001; Seki et al., 2001; Thimm et al., 2001; Kawaura et al., 2006; Ding et al., 2009; Zhu et al., 2013). Over the years, several experiments targeting to use of microarrays in drought-treated barley were also reported (Ozturk et al., 2002; Atienza et al., 2004; Guo et al., 2007; Talamè et al., 2007; Tommasini et al., 2008; Guo et al., 2009). However, most of these works operated with single genotypes (Ozturk et al., 2002; Atienza et al., 2004; Talamè et al., 2007; Tommasini et al., 2008), thus they lack a needful contrast between tolerant and susceptible cultivars, which is essential to differentiation of tolerance-related genes from those commonly responsive to drought. An issue connected with selected works (Ozturk et al., 2002; Atienza et al., 2004; Talamè et al., 2007) was also a fact, that authors often applied short dehydration intervals instead of longer drought periods, more characteristic for field conditions. On the other hand, experiments designed as multi-varietal (Guo et al., 2007, 2009) were oriented on latter developmental stages. Nevertheless, logical assumption would be, that barley plants are the most predisposed to drought in the early developmental stages, thus, monitoring of expression changes which come to progress after drought exposure should be targeted to these growth phases. What is general to the above mentioned works is the fact that neither of them evaluated the variation of expression profiles in different plant tissues of drought-treated plants, i.e., that DNA or RNA hybridized on microarray was isolated from only one plant tissue and almost exclusively from leaves (Ozturk et al., 2002; Guo et al., 2007; Talamè et al., 2007; Guo et al., 2009). In the studies focused on plant responses to drought stress at the gene level, changes have been neglected in crowns, a body which has for cereals significantly greater importance than individual leaves, because it differentiates the individual tillers and, unlike single leaves, it cannot be replaced. Importance to study transcript patterns alterations in crown was recently confirmed in experiments with cold and freezing stress (Winfield et al., 2010; Janská et al., 2011). However, until now, there is no manuscript reporting crown importance in coping with water stress.

In this article, we present a series of microarray experiments in an arrangement with two contrasting barley genotypes exposed to drought, where each genotype RNAs were isolated from leaves and crowns separately. This approach could bring completely new insight into barley drought defense mechanism via elucidation of crown role in drought survival.

## Materials and Methods

### Plant Material and Stress Treatment

**FIGURE 1:**
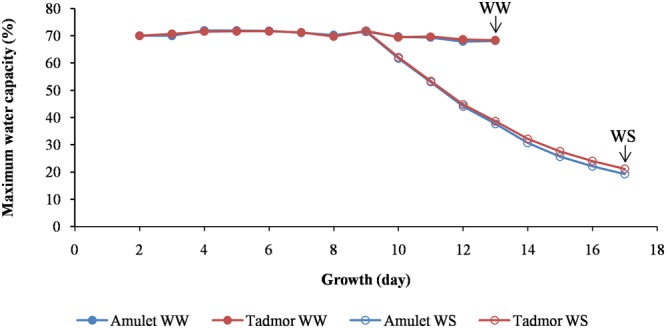
**Soil water content expressed relative to 100% maximum water capacity during the growth of two barleys (Amu and Tad).** In the ninth day of growth one-half of the pots were withheld from water for the next 8 days (WS). The arrows show the day of sampling plants from well-watered (WW) and water-stressed (WS) conditions which it was for both treatments when the plants reached the stage of a fully developed leaf 2.

The seeds of two barley (*Hordeum vulgare* L.) cultivars, Amulet (Amu; a spring barley variety, originating from the Czech Republic) and Tadmor (Tad; a two-row barley selected from the Syrian landrace Arabi Aswad), which are differentially sensitive to drought, were obtained from Mendel University in Brno (CZE). The seeds were germinated for 2 days at 24°C in the dark; then the seedlings were grown in pots (bottom diameter 13 cm, upper diameter 21 cm, height 20 cm) filled with soil (a mixture of Alfisol with manure and sand, 6: 2: 1) at 25°C/20°C (light/dark, 14 h/10 h, irradiation intensity 350 μmol⋅m^-2^⋅s^-1^ provided by a high-pressure sodium lamp + incandescent bulb) in a growth chamber (Tyler, type T-16/4, Budapest, Hungary) for the next 9 days. The soil was maintained at 70% of maximum water capacity (MWC), with watering of the pots each day to maintain a constant weight (5,500 g). Next, one-half of the pots were kept under the same conditions until the plants reached the stage of a fully developed second leaf (well-watered, WW). The remaining half of the pots were withheld from water for the next 8 days (water-stress, WS) until the plants had reached the same growth stage as in the WW treatment (**Figure 1**). When the plants reached the stage of a fully developed leaf 2, the five crowns and five leaf 2 were sampled from five plants grown in five different pots with the same treatment (WW or WS) for determination of water-relationship parameters in three repetitions. The same number of samples was taken for abscisic acid (ABA) determination as well as dehydrin protein analysis. The experiment was two times repeated. The plants (the second leaf and crown separately) were sampled from both conditions (WW and WS), in order to measure their water-relationship parameters. The plant tissues used for determination of ABA level, content of dehydrins, and transcription activities were snap-frozen in liquid nitrogen and stored at -80°C.

### Expression Profiling

The plant tissue was snap-frozen in liquid nitrogen and stored at -80°C, before being used for RNA extraction based on the TRIZOL reagent (Invitrogen, Carlsbad, CA, USA). The RNA was purified by passing through an RNeasy column in the presence of DNase (Qiagen, Hilden, Germany). RNA quality was assessed by both agarose gel electrophoresis and analysis in an Agilent 2100 Bioanalyzer (Agilent Technologies, USA). Each biological sample was represented by three independent replicates, each of which consisted of a bulk of four seedlings. Each RNA sample was hybridized to the Affymetrix 22 K Barley1 GeneChip Genome Array (Close et al., 2004). GeneChip^®^ hybridization quality was ensured by using standard controls supplied by manufacturer, and B2 oligonucleotides were added to each hybridization cocktail. PolyA controls (lys, phe, thr, dap) and hybridization controls (BioB, BioC, BioD and Cre) were used to monitor labeling and hybridization. Freely available software R (R Core Team, 2015) and associated library packages were employed for statistical computing. Raw data from microarrays were subjected to preprocessing analysis incorporating functions from Affy package library (Gautier et al., 2004), with emphasis on boxplots, density plots and Bland-Altman plot modification (MVA plot). Subsequently, Robust Multi-array Average method (RMA; Irizarry et al., 2003) from the same library was implemented for normalization. With the aid of this algorithm, background noise and processing artifacts were eliminated. Alongside, an iterative median polishing procedure summarized the data and generated a single expression value for each probe set. After normalization, repeated data verification was made by tools from initial analysis. Arrays exclusively meeting the criteria as recommended by manufacturer were submitted to further evaluation.

Differentially expressed genes (DEGs, |log_2_-fold|≥ 2) were identified via linear model for microarray analysis from Limma library package (Smyth, 2005). Using this model, pairwise analysis of individual samples against all others was accomplished, considering *P*-value < 0.05 as threshold of expression differences significance. Duplication discarding procedure provided reduction of redundant probe sets to unique records, which figured in repeated RMA as a restriction criterion. To reach comprehensive classification of mutual interactions across observed samples, expression values matching sorted probe sets were tested using principal component analysis (PCA) from a map library package (Lucas, 2014). Enhanced heat map (gplots library; Warnes et al., 2015) combined with hierarchical clustering was involved in manifesting disparity within RNA samples originated from drought-treated plants. Log2-transformed differences in transcription activity of described samples over parallel controls were color-scaled based on expression strength and clustered as measurement of similarity. In probe sets arising of aforesaid comparison, searching was done for numbers of genes specific or common to distinct stressed samples. Differentially expressed genes specific/common to singly treated samples was visualized using quad-set Venn diagrams (VennDiagram; Chen, 2013).

The linear model combined with moderate F statistics (Smyth, 2005) was used to identify genes with variety-specific response to drought, i.e., genes with a strong response to drought in one variety and low response in the second variety. The resulting variety-specific response was plotted as a scatter plot. Genes that do not respond to stress or respond similarly in both varieties are located in the origin, whereas variety-specific responding genes are located outside the origin.

Annotation of differentially expressed genes was mediated by Plexdb annotation tool. Consensus sequences of selected probes were blasted against Uniref90 (last release) using blastx with maximum *e*-value of 1e-4.

### Water Content, Water Saturation Deficit, and Osmotic Potential Assessment

The water content (WC, % of water weight to fresh weight) was measured in the crowns (under-ground bases (nodes) of plants), and the second leaves for both treatments. Moreover, the water saturation deficit (WSD) and osmotic potential (OP) were measured in leaf 2. OP was determined psychrometrically from the leaf sap, using a Wescor HR-33 microvoltmeter (USA) with C52 sample chambers. Results were expressed in MPa, determined by reference to calibration solutions of NaCl. WSD was determined in cut leaf segments (1 cm length) which were rapidly weighted (FW), then water-saturated in a wet sheet of foam, and weighted after 3 h (W3) and 6 h (W6), and finally after drying at 90°C (DW). WSD was calculated by Slavík (1974): WSD (%) = [(2W3 – W6 – FW)]/[(2W3 – W6 – DW)] × 100.

### Abscisic Acid and Assessment of Dehydrin Proteins

The samples for (±) ABA analysis were homogenized and extracted into distilled water (0.1 g FW/1 ml H_2_O), shaken for 16 h under cold (4–5°C) and dark conditions, and processed by indirect ELISA according to Asch (2000). For each sample, we used three replicates on a microtitre plate. An extinction photometer SUNRISE Remote (Tecan, Germany) was used to measure color intensity of the final product at 405 nm, and the ABA concentration was calculated.

The dehydrin extraction as well as the 1D SDS-PAGE and immunoblots analyses were performed according to Vítámvás et al. (2007). Proteins were separated by SDS-PAGE on 10% gels (Laemmli, 1970; Multigel-Long, 24 well comb, Whatman Biometra). About 3 μg of soluble proteins (upon boiling) extracted from crowns and leaves were loaded on gels. To compare intensities of different samples on different membranes, calibration samples with different load (cca 1:5 of crown tissue) were added on gels as well. Precision Plus Protein All Blue Standards, mixture of ten blue-stained recombinant proteins (10, 15, 20, 25, 37, 50, 75, 100, 150, 250 kDa) were also added to gels. Gels were stained by Bio-Safe Coomassie (Bio-Rad) to obtain sample load controls. The relative accumulation of dehydrins was determined densitometrically using Quantity One software (Bio-Rad, v. 4.6.2., Munich, Germany).

### Statistical Analysis (WC, WSD, OP, ABA, and Dehydrin Proteins)

Results were expressed as averages calculated from six repetitions, and compared using a *T*-test, ANOVA analysis, and Duncan’s multiple range test at the 0.05 level (Statistica v. 10 software, StatSoft, Tulsa, OK, USA).

## Results

### Water Content, Water Saturation Deficit, and Osmotic Potential

**FIGURE 2:**
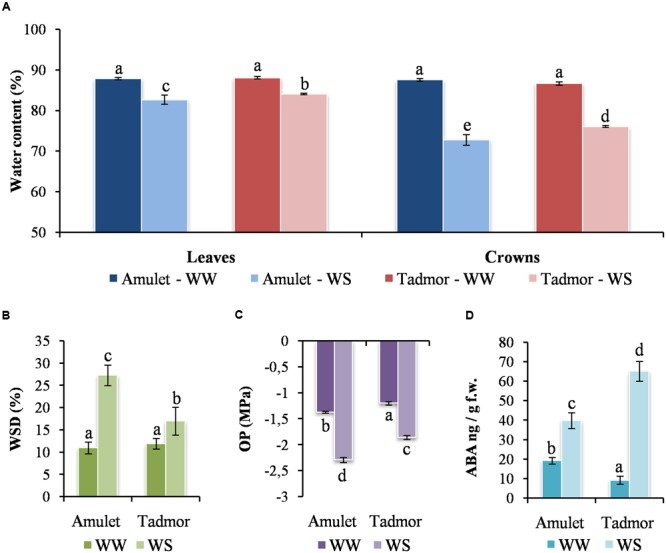
**(A)** Water content (WC) in leaf and crown, **(B)** water saturation deficit (WSD), **(C)** osmotic potential (OP) and **(D)** abscisic acid content (ABA in leaves of two barleys (Amu and Tad) from well-watered (WW) and water-stressed (WS) conditions. Error bars represent mean ± standard deviation. The means with the same letters above the bar are not significantly different.

Water content significantly decreased in all parts of plants under WS (water stress) conditions (**Figure 2A**). Reduction of WC was greater in the crowns than in the leaves. However, Tad retained significantly higher WC in the leaves and crowns than Amu did in WS plants. WSD increased and OP decreased in the second leaf in both varieties after WS conditions. WSD was higher and OP was lower in Amu than in Tad (**Figures 2B,C**).

These results showed that both varieties reached water scarcity under WS conditions. However, Tad lost less water and suffered lower water deficit than Amu.

### ABA Content and Dehydrin Accumulation

The content of ABA significantly increased in both varieties under WS conditions. The level of ABA increased much more in Tad than Amu, although its lower content in Tad control plants (**Figure 2D**) indicating the important signaling role of ABA in the adaptation to drought stress.

Among DEGs, two were presented that encode key enzymes participating in ABA biosynthesis, 9-*cis*-epoxycarotenoid dioxygenases NCED9 (*at1g78390.1)* and NCED4 *(at4g19170.1)*. These two genes, however, show different trend of expression (see **Table 1**). *NCED9* significantly increased its expression in leaves of both genotypes with Amu having the higher level, while *NCED4* lowered its expression in leaves of both genotypes and such decline was stronger in Amu. Although in both cold and drought stress ABA seems to play a central role in the basal stress response, the mechanisms which ABA is regulated might differ. For example the *NCED4* gene was up-regulated in cold stress treatment in our previous study (Janská et al., 2014).

**Table 1:** Differentially expressed genes (DEG)s encoding ABA biosynthetic enzymes.

ID^a^	Log2 FC^b^				Affymetrix annotation^c^	AGI^d^
	Amuleaf	Amucrown	Tadleaf	Tadcrown		
HT11N18r_s_at	3.548	0.654	1.106	-0.281	viviparous-14 protein	*at1g78390.1*
Contig4988_at	-2.232	0.049	-1.151	0.445	9-*cis*-epoxycarotenoid dioxygenase	*at4g19170.1*

*^a^Affymetrix 22 K Barley1 GeneChip Genome Array probe ID. ^b^Log2 transformed fold change of treated samples against parallel controls. ^c^Microarray manufacturer (Affymetrix) annotation of individual IDs. ^d^*Arabidopsis* locus identifier corresponding to individual IDs.*

**FIGURE 3:**
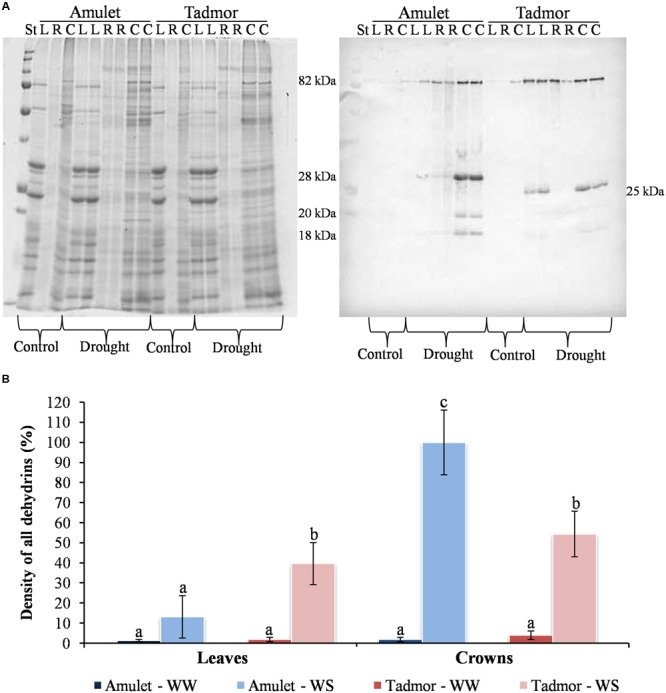
**(A)** Coomassie stained gel (left) and protein gel blot (right) showing proteins soluble upon boiling and dehydrin protein accumulation, respectively. Proteins were separated by SDS-PAGE on 10% gels [11 × 12 cm (w × l), 24 well comb]. About 3 μg of crown (C) and leaf (L) proteins soluble upon boiling were loaded on the gels. St represents the Precision Plus Protein All Blue Prestained Protein Standards, mixture of ten blue-stained recombinant proteins (10, 15, 20, 25, 37, 50, 75, 100, 150, 250 kD). R represents calibration samples (1:5 of crown tissue) for comparison intensities of different membranes. **(B)** Dehydrin protein density in leaf and crown of two barleys (Amu and Tad) from well-watered (WW) and water-stress (WS) conditions. Error bars represent mean ± standard deviation. The means with the same letters above the bar are not significantly different.

Immunoblot analysis of leaf and crown samples revealed five detectable dehydrin bands of different molecular weights: 82, 28, 25, 20, and 18 kDa (**Figure 3A**). In all samples, the most abundant dehydrin protein was 80 kDa, corresponding to dehydrin 5 (Kosová et al., 2010). The composition of individual dehydrins was different in the two varieties. For example, the 25 kDa dehydrin was characterized in Tad, whereas the 28 kDa dehydrin was found in Amu. The relative content of dehydrins was higher in the crowns than in leaves in both varieties, and the density of all dehydrins was higher in the crowns of Amu than those of Tad in WS conditions (**Figure 3B**).

In treated barley plants we identified 16 genes encoding dehydrins which compared to the non-treated plants were significantly expressed (see **Table 2**). Based on the expression changes, we can conclude that in the crowns Amu exhibited higher expression of dehydrin-encoding genes than Tad did. However, significant difference between the cultivars was recorded only for genes encoding dehydrins 5 (*at3g50970.1*) and 10 (*at3g50970.1; at2g21490.1*). Tad exhibited higher expression of certain dehydrin-encoding genes in leaves compared to Amu.

**Table 2:** Differentially expressed genes encoding dehydrins.

ID^a^	Log2 FC^b^				Affy.anot.^c^	Structural type^d^	AGI^e^
	Amu leaf	Amu crown	Tad leaf	Tad crown			
Contig1701_s_at	6.187	7.436	6.826	5.989	Dhn 2	*Y_n_SK_m_*	*at3g50980.1*
Contig1721_at	5.218	7.554	6.159	6.132	Dhn 2	*Y_n_SK_m_*	*at5g66400.1*
Contig1724_s_at	8.082	7.589	8.091	6.984	Dhn 3	*Y_n_SK_m_*	*at5g66400.1*
Contig1713_s_at	7.854	8.426	7.693	7.071	Dhn 4	*Y_n_SK_m_*	*at5g66400.1*
Contig1717_s_at^∗^	0.622	3.228	1.363	1.111	Dhn 5	*K_n_*	*at3g50970.1*
HVSMEa0006I22r2_s_at	0.393	4.775	2.036	3.699	Dhn 5	*K_n_*	*at3g50970.1*
Contig1708_s_at	0.311	2.767	0.261	3.113	Dhn 6	*Y_n_SK_m_*	*at4g01985.1*
Contig1709_at	7.493	7.551	7.307	6.527	Dhn 7	*Y_n_SK_m_*	*at5g66400.1*
Contig1725_s_at	7.367	9.022	7.305	7.168	Dhn 8	*SK_n_*	*at5g66400.1*
Contig2855_at	1.159	0.763	0.772	-0.504	Dhn 8	*SK_n_*	*at1g20440.1*
Contig1718_s_at	5.163	5.324	4.024	4.247	Dhn 9	*Y_n_SK_m_*	*at3g50980.1*
Dhn10(Morex)_s_at^∗^	6.473	6.989	4.208	3.790	Dhn 10	*Y_n_SK_m_*	*at3g50970.1*
Contig13753_at^∗^	5.343	6.502	3.499	2.955	Dhn 10	*Y_n_SK_m_*	*at2g21490.1*
Contig10207_s_at	0.704	1.644	-0.139	1.076	Dhn 11	*Y_n_SK_m_*	*at5g66400.1*
Dhn12(Morex)_at	-0.176	-0.197	1.698	0.573	Dhn 12	*Y_n_SK_m_*	*at5g66400.2*
Contig15845_s_at	0.131	0.136	1.611	0.195	Dhn 12	*Y_n_SK_m_*	*at5g66400.2*

*^a^Affymetrix 22 K Barley1 GeneChip Genome Array probe ID. ^b^Log2 transformed fold change of treated samples against parallel controls. ^c^Microarray manufacturer (Affymetrix) annotation of individual IDs. ^d^Structural type of individual dehydrins. ^e^*Arabidopsis* locus identifier corresponding to individual IDs.*

### Global Comparison of Expression Profiles

To investigate mutual interactions within dataset, pairwise analysis of every sample combination was performed followed by PCA of RMA subjected expression values matching DEGs (|log_2_-fold|≥ 2, *P* ≤ 0.05). As is evident from PCA (**Supplementary Figure 1**) the distinction of biological replicates within individual samples represents a smaller component of total variance than variability across observed samples, thus adding further confirmation of data quality.

### Differentially Expressed Genes (DEGs)

**FIGURE 4:**
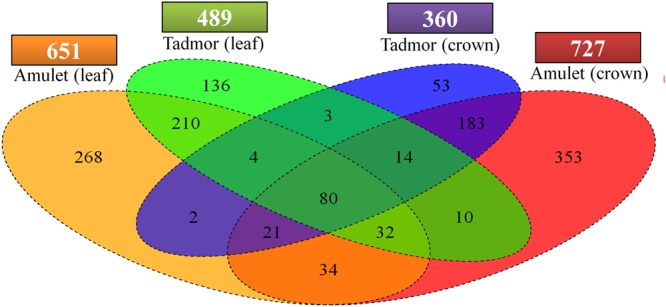
**Quad-set Venn diagram displaying numbers of genes, which were differentially transcribed between control and drought stress condition.** Overlapping, exclusive regions in diagram correspond to DEGs common or specific to single treated samples. Numbers above sample names represents total tally of DEGs recorded in single treated samples on chosen level of significance.

**FIGURE 5:**
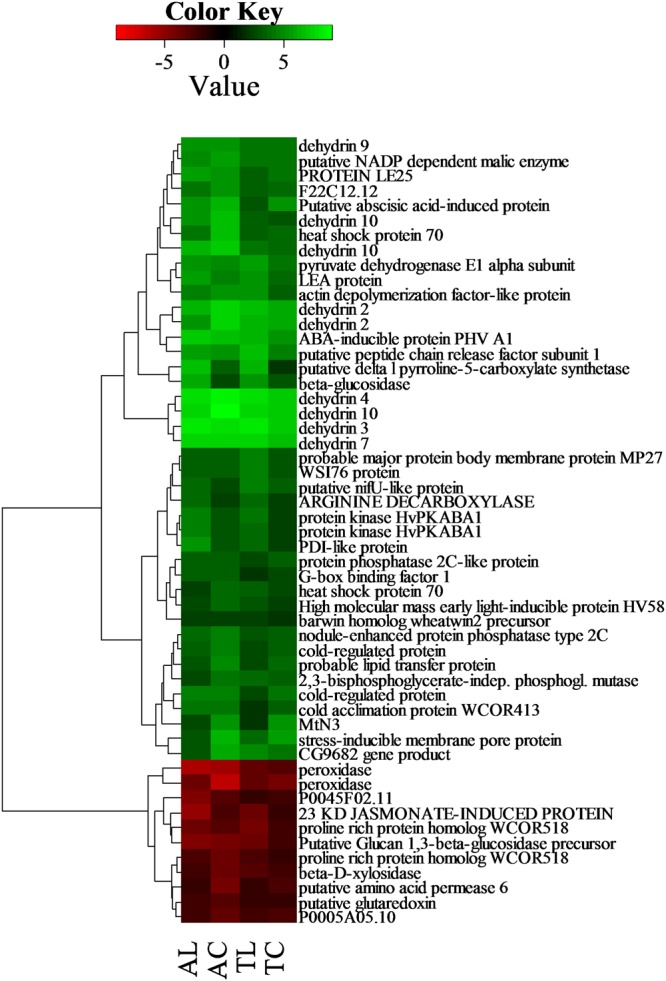
**Expression disparity and cluster analysis of genes, which were differentially expressed between control and drought stress condition.** DEGs common to all treated samples were used, unknown, and hypothetical proteins were excluded from analysis. The color saturation reflects the fold change as visualized in color key. T is Tadmor, A is Amulet, L is leaf and C is crown.

Susceptible variety Amu exhibited higher number of DEGs as a response to drought stress (**Figure 4**). Moreover, Amu has responded to stress more strongly in crown tissue (727) than in the leaf tissue (651), while Tad has been characterized by an inverse relationship (leaf – 489, crown – 360). Eighty DEGs were common to all treated samples (**Figure 5**).

#### DEGs Common to All Treated Samples

Of the 80 DEGs common to all treated samples, [65 up-regulated, 14 down-regulated, 1 inversely regulated (**Figure 5**, Supplementary Table 1)], there we found four overrepresented groups: those DEGs associated with ABA (17 genes), water deprivation (11 genes) and osmotic stress (16 genes) and those encoding lipid transfer proteins (LTPs, 8 genes) that point out to the central role of ABA and osmotic regulation in the face of drought stress independent of the genotype tolerance level.

Abscisic acid is the key mediator of dehydration signaling and is also involved in responses to other abiotic stress response pathways including salt stress or cold (e.g., Ding et al., 2013). This is true mainly for group of genes encoding dehydrins strongly up-regulated and overrepresented in this category, esp. DHN5, 7 and 10 as annotated for barley [DHNs 5, 2, 3 (*at5g66400.1*) and DHN1 (*at3g50970.1*) as annotated for *Arabidopsis thaliana*]. Among DEGs commonly up-regulated in all treatments and genotypes, P5CS (delta1-pyrroline-5-carboxylate synthase 1, *at2g39800.4*) important for proline biosynthesis or ADC (*at4g34710.2*, arginine decarboxylase 2) involved in the first step of polyamine synthesis were represented. Down-regulation of aquaporins (*at3g53420.2*) is an important step in water retention.

From the group of LTPs, the most overrepresented LTP4 (*at5g59310.1*) is also strongly induced by ABA and localized to the cell wall.

The other two overrepresented functional categories, osmotic stress and water deprivation combines genes from both the ABA-regulated and LTPs groups, excluded genes coded for ABA signaling pathway inhibitors NF-X-like 1 (*at1g10170.1*) and protein phosphatase 2CA (PP2C, *at3g11410.1*). PP2C hub allows the coordinated activation of ABA and energy signaling, strengthening the stress response through the cooperation of two key and complementary pathways (Rodrigues et al., 2013).

Protein phosphatase 2C (PP2C) is known to act antagonistically to MAPKKK kinase cascade and SnRK involved in ABA-mediated signaling and signal transduction from plasmalemma to nucleus. It is known that ABA receptor PYR1 activated by ABA inhibits PP2C (Park et al., 2009). Differential phosphorylation of PP2C under drought with respect to control conditions was found in drought-treated wheat (Zhang et al., 2014).

#### Differentially Expressed Genes Responding Differently in the Tolerant Variety Relative to the Susceptible Variety

**FIGURE 6:**
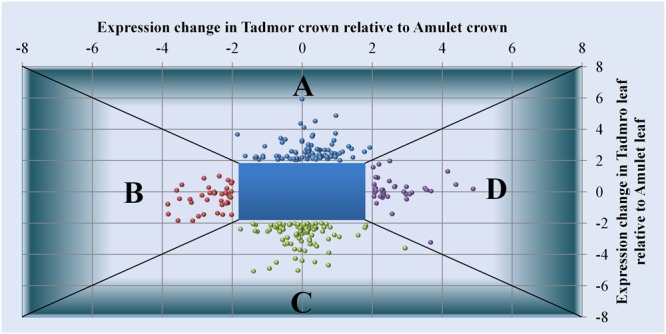
**Genes differentially expressed in Tad relative to Amu: (A)** enhanced expression in leaf, **(B)** reduced expression in crown, **(C)** reduced expression in leaf **(D)** enhanced expression in crown. Horizontal axis corresponds to expression values of individual genes in crowns, while on vertical axis, there are expression differences recorded in leaves.

Using the linear model in combination with moderated F statistics we identified DEGs responding differently in the tolerant genotype relative to the susceptible variety (**Figures 6A–D**). We presume that modulated expression of such genes is a factor, which provides the tolerant cultivar a competitive advantage over the susceptible one under water scarcity.

#### Genes Whose Expression Is Enhanced in Tad Relative to Amu in Leaves

First group of 90 DEGs (28 unknown) covers probe sets whose expression is enhanced in Tad relative to Amu in leaf tissue (**Figure 6A**, Supplementary Table 2). These genes are profusely represented by those encoding various classes of heat shock proteins [HSPs: 2x HSP17.9 (*at5g59720.1*), 6x HSP17.8 (*at5g12020.1*), 2x HSP40 (*at3g44110.1, at1g56300.1*), 2x HSP70 (*at3g12580.1*), 2x HSP80 (*at5g56010.1*), 1x HSP100 (*at2g25140.1*)]. Small HSPs as well as HSP70 and HSP100 are well known as drought-responsive (Ristic et al., 1998; Grigorova et al., 2011a,b). We also detected gene encoding TPR-repeat protein (*at1g62740.1*), which is an important component of protein–protein interactions and coordinates HSP70 and HSP90 co-chaperone activity (Song and Masison, 2005).

Another genes represented encode proteins similar to zinc finger protein (*at5g64920.1*) and myb-related protein (*at4g39250.1*). Expression of these proteins frequently followed water deprivation and sometimes they alleviated drought impact or conferred desiccation tolerance (Martin and Paz-Ares, 1997; Takatsuji, 1998; Mukhopadhyay et al., 2004; Cominelli et al., 2005; Dai et al., 2007; Xu et al., 2008; Ding et al., 2009; Qin et al., 2012).

Among genes enhanced in Tad relative to Amu in leaves, there were also those encoding enzymes acting in sulfur metabolism (adenosine 5′-phosphosulfate reductase (APR; *at4g04610.1*), cysteine synthase (CSase; *at3g22460.1*), homocysteine *S*-methyltransferase-3 (HMT-3; *at3g63250.1, at3g63250.2*). Tad also achieved higher expression level of genes encoding proteins related to lipid biosynthesis or transport as well as to cutin formation (GDSL-motif lipase/hydrolase-like protein; protein (GDSL; *at5g55050.1*), lipid transfer protein (LTP; *at4g12480.1*). It was proved that changes in lipid composition may help to maintain membrane integrity and preserve cell compartmentation under water shortage (Blum and Ebercon, 1981; Quartacci et al., 1995; Aziz and Larher, 1998; Toumi et al., 2008). A positive relation between cutin content and drought tolerance was also observed (Yang et al., 2011).

Higher transcription activity of genes encoding purple acid phosphatase (PAP; *at1g52940.1*) and ferritin (*at3g56090.1*) in Tad can be credited to their ROS scavenging activities. PAP from *Arabidopsis* plants was described to accumulate in response to phosphate starvation, ABA, salt stress and oxidative stress (del Pozo et al., 1999). In addition, it was confirmed that mitochondrial PAP reduced ROS, which alleviated osmotic stress in soybean plants (Li et al., 2008). Increased ferritin activity followed after drought exposure was also described (Jiang et al., 2012; Xu and Huang, 2012; DeLaat et al., 2014).

Better ability to cope with water scarcity could be also credited to an over-expression of probe sets encoding receptor protein kinase (*at3g51550.1*), or peptidyl-prolyl *cis-trans* isomerases (PPs FKBP77 (*at3g25230.2*), which were described as drought responsive (Guo et al., 2009). In leaves, Tad also effectively employed genes encoding glutamate decarboxylase (GAD; *at2g02000.1*) and anthranilate-synthase (AS) alpha 1 subunit (*at3g55870.1*). GAD drives a conversion of glutamic acid to γ-aminobutyric acid. Experiment with two contrasting wheat cultivars showed an importance of this non-protein amino acid for desiccation tolerance acquisition (Saeedipour and Moradi, 2012).

#### Genes Whose Expression is Reduced in Tad Relative to Amu in Leaves

Within a group of genes whose expression was reduced in Tad relative to Amu in leaves [95 DEGs (30 unknown), **Figure 6C**, Supplementary Table 4], there were those encoding jasmonate induced-proteins [unspecified JIP (*at3g51430.2*) and JIP60 (*at5g01280.1*)]. JIP60 is involved in translation regulation or processes such as leaf senescence or programmed cell death (Sembdner and Parthier, 1993; Chaudhry et al., 1994).

Co-inhibition of phenylalanine ammonia-lyase (*at2g37040.1*), chalcone synthase (*at5g13930.1*) and bZIP protein HY5 (*at5g11260.1*) by Tad lead us to a suggestion about anthocyanins accumulation blocking in Tad or its stimulation in Amu. Tad also showed reduced expression of genes encoding proteins connected to secondary cell wall properties [cellulase (*at3g44990.1*), cellulase synthase OsCsIE1 (*at1g55850.1*), expansin (*at1g69530.2*), *O*-methyltransferase ZRP4 (OMT ZRP4; *at4g35160.1*)].

In a group of leaf specific transcripts whose expression is reduced in Tad relative to Amu, there are several genes encoding photosynthesis-related proteins [chlorophyll a/b WCAB precursor (*at2g34420.1*), chlorophyll a/b protein 25 (*at2g34420.1*), light-inducible protein CPRF-2 (*at5g28770.2*)] as well as genes encoding gibberellin and cytokinin biosynthesis enzymes [kaurene synthase (*at1g79460.1*) and cytokinin oxidase (*at1g75450.1*)].

#### Genes Whose Expression is Enhanced in Tad Relative to Amu in Crowns

Among genes whose expression was enhanced in Tad relative to Amu crowns (40 DEGs (13 unknown), **Figure 6D**, Supplementary Table 5), the most abundant were transcripts encoding jasmonate induced proteins [JIPs; 32.6 kDa JIP (*at1g19715.3*), 32.7 kDa JIP (*at1g19715.3*), 23 kDa JIP, unspecified JIP, thionin (*at1g66100.1*), thionin Osthi (*at2g15010.1*), 12-oxophytodienoate reductase (OPR; *at1g76680.1*)]. Jasmonates is a group of plant hormones widely influencing physiological and developmental processes within plants. In addition, they perform a key role in response to stress of both biotic and abiotic nature (Santino et al., 2013). In plants, jasmonates can induce *de novo* synthesis of proteins (JIP – jasmonate induced proteins).

Similarly to leaves, even within crown tissue, there were overrepresented transcripts encoding proteins involved in or connected to cell wall properties [(LTP; *at4g12500.1*), caffeic acid *O*-methyltransferase (COMT; *at5g54160.1*), shikimate kinase (*at2g21940.5*)]. A higher number of transcripts encoding these proteins within crown suggests, that tissue tolerance relies on cell wall modification processes. As noted, LTPs proteins participate not only in lipid transport, but play an important role in cutin digestion as well. This polymer represents a matrix for a lipophilic barrier and ensures various functions in plants (Pollard et al., 2008). Cutin beneficial effects on drought tolerance have been described previously (Chen et al., 2011; Seo and Park, 2011; Al-Abdallat et al., 2014).

Crowns of tolerant genotype also showed high transcriptional activity of gene encoding DNA-binding protein ABF2 (*at1g80840.1*), a basic domain/leucine zipper transcription factor that cooperatively regulates ABRE-dependent ABA signaling involved in tolerance to drought and other stresses (Kim et al., 2004; Fujita et al., 2005; Yoshida et al., 2010). ABF2 and other master transcriptional factors are induced by dehydration, high salinity, or ABA treatment in vegetative tissues (Fujita et al., 2005), and their gain-of-function mutants showed lower drought stress tolerance (Kim et al., 2004; Fujita et al., 2005).

In the crown of Tad, there was also the higher level of genes encoding proteins associated with nucleosome remodeling [Histone H3 (*at5g65360.1*), H4 (*at5g59970.1*)].

### Genes Whose Expression is Reduced in Tad Relative to Amu in Crowns

Among crown-specific transcripts whose expression is reduced in Tad relative to Amu [39 DEGs (13 unknown), **Figure 6B**, Supplementary Table 3], there were some genes encoding HSPs such as HSP17 (*at5g59720.1*), HSP18 (*at5g59720.1*), HSP70 (*at3g12580.1*) and dehydrins [DHN 5 (*at3g50970.1*), DHN10 (*at3g50970.1, at2g21490.1*)] as well as genes encoding enzymes involved in carbohydrate metabolism [WSI76 (*at1g09350.1*), sucrose synthase 2 (SuSy2; *at3g43190.1*)]. Another gene reduced in Tad relative to Amu encodes cytosolic aldehyde dehydrogenase RF2C (RF2C; *at3g24503.1*). This cytosolic aldehyde dehydrogenase is involved in a biosynthesis of ferulic acid, which has been documented to respond positively to drought stimuli and to be more accumulated in drought tolerant genotypes (Hura et al., 2007, 2008, 2009).

Lower transcription activity of genes encoding some proteins with transcription regulation activities, such as MADS box protein 5 (*at1g69120.1*) and methyl binding protein MBD108 (*at5g35330.3*) were also detected in Tad crowns (**Figure 6B**, Supplementary Table 3). Transcriptional regulators are an important component of plant response to abiotic stresses including drought. Nevertheless, wide scale study of genes of MADS-box family showed that some representatives are up-regulated, while others are down-regulated upon drought (Arora et al., 2007).

## Discussion

Drought tolerance is a very complex trait that relies on the inherent ability of the plant to sustain growth (likely at a reduced rate) even when the conditions are unfavorable for the maintenance of basic plant processes (Peleg et al., 2011) and includes series of physiological and biochemical responses (Cattivelli et al., 2008). Therefore, creating tolerant genotypes is great challenge for genetic engineering (Cattivelli et al., 2008). Our goal was to bring the comprehensive overview of the most important processes in barley water stress coping strategies and to find out the role of crowns in barley drought stress response.

Our results point out the central role of ABA and osmotic regulation in the face of drought stress independent of the tissue and genotype tolerance level in the transcriptomic and proteomic level as well as on the ABA level in barley leaves and crowns. ABA is the key mediator of dehydration signaling and is also involved in responses to other abiotic stress response pathways including salt stress or cold (e.g., Ding et al., 2013). This is true mainly for group of genes encoding LEA (late embryogenesis abundant) proteins including dehydrins strongly up-regulated in the susceptible as well as tolerant cultivar. These proteins are well documented to accumulate in plants affected by water scarcity, where they prevent conformation changes of other proteins that could lead to loss of their activity and under most severe drought also to their denaturation or aggregation (Goyal et al., 2005). LEA proteins accumulate in response to drought in all vegetative parts of the plant, so it is not surprising that in our experiment, LEA proteins encoding genes were strongly induced by stress in both investigated tissues. However, the fact that some LEA protein-encoding genes were strongly accumulated in leaf, while others in crowns supports the hypothesis about importance of both leaves and crowns in water deficit coping strategies. Dehydrin genes including high-molecular Kn type dehydrin 5 (DHN5) as well as low-molecular YxSKn type dehydrins found in immunoblots (protein gel blots) are induced by ABA. Although Tad revealed higher levels of ABA, dehydrin protein accumulation was lower in Tad than in Amu.

Genes commonly responsive to drought were also represented by those encoding HSP70 and PDI-like protein, which participate in protein folding processes, including prevention of aggregation of proteins into large potentially cytotoxic complexes. Similarly to LEA, HSP70 and PDI-like protein encoding genes were numerously observed to accumulate under dehydration (Cho and Hong, 2006; Cho and Choi, 2009; Guo et al., 2009; Zhu et al., 2014; Augustine et al., 2015). From the group of LTPs, the most overrepresented LTP4 expressed differentially in all samples, is also strongly induced by ABA and localized to the cell wall. LTP4 is predicted to be a member of PR-14 pathogenesis-related protein and involved in a wide range of abiotic stress responses (Gao et al., 2015). Down-regulation of aquaporins is an important step in water retention. This observation is similar to our previous experiments with cold treatments in barley (Janská et al., 2014).

Among DEGs commonly up-regulated in all treatments and genotypes, ADC, involved in the first step of polyamine synthesis, P5CS important for proline biosynthesis or gene encoding galactinol synthase, were represented. It has been observed, that expression of ADC in drought treated plants led to several positive changes, including membrane stabilization, an increase in free proline content, an improvement of water use efficiency and net photosynthesis and other processes that enhanced plant tolerance of affected plants (Farooq et al., 2009). P5CS is linked to a biosynthesis of an amino acid proline. Similarly to polyamines, proline also acts in various tasks within drought-treated plants, including photosynthesis (Szabados and Savoure, 2010). WSI76 is thought to encode galactinol synthase, which catalyzes the first step in the biosynthesis of raffinose family oligosaccharides (RFO) and has been found out to accumulate in seed and vegetative part of plants after desiccation (Liu et al., 1998; Taji et al., 2002; Li et al., 2011; Sun et al., 2013). Genes encoding WSI76 were shown to reach the highest expression level in leaf tissue of Tad (**Figure 5**, Supplementary Table 1).

Our results point out the exceptional role of ABA in coping with drought stress independent of tissue type and tolerance level as well as important role of genes encoding proteins induced by ABA and involved in osmotic adjustment such as LEA proteins and genes encoding osmolyte biosynthesis enzymes.

### Genes Whose Expression is Reduced/Enhanced in Tad Relatively to Amu

There are some Tadmor specific transcriptional responses to drought stress that could be the important components of the complex response of the high drought stress tolerant cultivars. Tadmor leaves remarkably enhanced expression of various classes of heat shock proteins after drought treatment. Results of some papers suggest that some HSPs could represent a possible source of drought tolerance. For example, Grigorova et al. (2011a) monitored an effect of combined drought-heat stress on the expression of selected HSPs in two contrasting wheat cultivars and detected a higher accumulation of smHSPs in drought tolerant genotypes. In addition, Guo et al. (2009) in large scale expression profiling of three variously drought-resistant cultivars, registered HSP17.8 as a specific response of tolerant genotypes. On the contrary, authors recorded HSP17.9 in both tolerant and susceptible cultivar. Similarly, HSP70 was reported to confer drought tolerance in plants (Cho and Choi, 2009). However, HSP70s are a variable group and not all representatives are drought-responsive (Sarkar et al., 2013). In barley, the gene encoding this protein was induced in both tolerant and susceptible genotype (Guo et al., 2009). HSP80 was described to create complexes with HSP70 in *Neurospora crassa* (Ouimet and Kapoor, 1998) and, as such, they could be relevant in drought defense. Presence of HSP40s (DnaJ) could be credited to their co-activity with HSP70s and their capability to drive functional specificity of HSP70s (Cyr et al., 1994). However, some papers also suggest HSP40s significance for photosynthetic reactions (Chen et al., 2010). Thus these proteins could significantly contribute to drought tolerance. We also detected enhanced expression of gene encoding TPR-repeat protein, which is an important component of protein–protein interactions and coordinates HSP70 and HSP90 co-chaperone activity (Song and Masison, 2005). Increased TPR activity upon drought-stress is well documented (Rosado et al., 2006; Zhu et al., 2013).

Our results support also the statement of Chan et al. (2013) that certain steps in sulfur metabolism are of great importance in drought stress signaling and response. Based on the above, we deduced that one of the defense strategies of tolerant cultivar facing water scarcity is an improved utilization of sulfur.

It was proved that changes in lipid composition could help to maintain membrane integrity and preserve cell compartmentation under water shortage (Blum and Ebercon, 1981; Quartacci et al., 1995; Aziz and Larher, 1998; Toumi et al., 2008) as well as a positive relation between cutin content and drought tolerance (Yang et al., 2011). Because Tad also achieved higher expression level of genes encoding proteins related to lipid biosynthesis or transport as well as to cutin formation, we suggest that increased activity of GDSL as well as LTPs in response to drought could be another mechanism of tolerant cultivar to cope with water scarcity.

The altered lipid metabolism is probably important drought stress tolerance mechanism not only in leaves, but also in the crowns. Besides gene encoding LTPs, two transcripts encoding caffeic acid *O*-methyltransferase (COMT) were presented. COMT was recently described as modulator of lignin content and composition (Guo et al., 2001). There are also some evidences about COMT contribution to drought tolerance (Hu et al., 2009). Shikimate kinase, which expression was also enhanced in Tad crowns after drought stress, plays a pivotal role in the formation of aromatic secondary compounds in plants and finally leads to the formation of, for example, stilbenes, flavonoids, and lignins. Considering the above, our assumption is that tolerant genotype efficiently utilizes and modulates lignin biosynthesis. Such effort may result from an endeavor to preserve tissue itself. Second alternative is that crown alters lignin in order to strengthen roots endurance. Observed roots lignification upon drought in several thesis supports such hypothesis (Cruz et al., 1992; Vartanian et al., 1994).

Tad crowns enhanced expression of genes connected to jasmonate signaling such as small JIPs (JIP 32.6, JIP 32.7, JIP 23), OPR and thionins. JIP23 is the most abundant barley JA induced protein and was reported to be localized specifically in highly osmotically stressed cells (Hause et al., 1996). These findings together with our results suggests its role in drought-stress response of plants. OPR is an enzyme directly involved in jasmonate biosynthesis and was recorded to enhance osmotic and salt stress tolerance in *Arabidopsis* plants during seed germination (Gu et al., 2008). Thionin represents one of the main jasmonate-induced proteins usually documented as a response to biotic stresses (Bohlmann et al., 1988; Carmona et al., 1993; Molina et al., 1993; Epple et al., 1997; Muramoto et al., 2012). Nevertheless, *thionin* up-regulation after drought exposure was described as well (Ozturk et al., 2002).

In the crowns of Tad, there we found out enhanced expression of genes encoding proteins associated with nucleosome remodeling (histone H3 and H4). The altered expression of such genes was described to be mechanism of barley freezing and cold tolerance. Since the gene expression of such genes was altered exclusively in the crown (Janská et al., 2011, 2014) and because freezing similar to drought causes cell dehydration, we conclude that differential expression of histones encoding genes might be also one of the mechanisms that enables Tad to cope with water stress.

Our results provide support to the proposal made by Fowler and Thomashow (2002) that the selective repression of genes is likely to represent a major component of the acclimation response. We suggest this implication also for drought stress. This is probably true for groups of genes promoting growth and genes encoding proteins involved in programmed cell death or senescence in the face of drought stress. Such genes could help the tolerant genotypes to redirected the energy toward stress adjustment mechanisms and prevent leaf senescence or death. So, Tad negatively regulated genes encoding enzymes involved in biosynthesis of gibberellin and cytokinin, phytohormones inducing cell division and growth. That suggests expression of the genes involved in active growth and development in Amu during drought stress. On the other hand, Tad facing drought lowered transcription of such genes in order to survive. Endeavor of Amu for active growth facing drought is supported by higher activity of pseudo-response regulator (PRR; *at5g60100.2*) encoding gene. The PRR is a part of regulatory pathway, which (via *CONSTANS* and *VRN3/FT1* genes) can induce transition from the vegetative to the reproductive phase under long day.

Also decreased transcription activity of genes for cellulase, cellulase synthase and expansin indicates reduced cell wall elongation in Tad contrary to Amu and therefore cessation of plant growth in Tad. Some negative effect on plant growth could also have a gene encoding NAC6 (*at1g01720.1*; **Figure 6C**, Supplementary Table 4). This transcriptional factor was described to induce drought tolerance (Nakashima et al., 2007, 2012). However, such improvement was redeemed by a retarded growth and a lower productivity of plants.

Drought leads to a repression of many genes related to photosynthesis at transcriptional level (Chaves et al., 2009). This is true also for our results. Repression of such genes may also resulted from photosynthetic accommodation strategies of Tad, such as reduced chlorophyll content (Tardy et al., 1998; Havaux and Tardy, 1999).

Higher transcription of genes encoding JIP60 along with genes for bowman-birk type trypsin inhibitor (*at2g40070.2*), subtilase (*at5g11940.1*) and subtilisin-like proteinase (*at5g11940.1*), in Tad relative to Amu (**Figure 6C**, Supplementary Table 4) indicates leaf senescence or cell death processes in susceptible genotype or their absence, possibly delaying in the tolerant one. Delaying of leaf senescence was described as a mechanism of drought tolerance acquisition (Rivero et al., 2007; Muchero et al., 2013). Leaf senescence is also related to the changes in peroxidase activities (Kar and Mishra, 1976). So, a diminution of peroxidase (*at5g05340.1*) expression in Tad relative to Amu (**Figure 6C**, Supplementary Table 4) could be explained by an ability of resistant genotype to avoid or postpone such drought consequences. On the other hand, peroxidase repression in Tad can be a part of an oxidative burst blocking or wilting precluding initiative (Lagrimini et al., 1990).

Co-inhibition of phenylalanine ammonia-lyase, chalcone synthase and bZIP protein HY5 by Tad lead us to a suggestion about anthocyanins accumulation blocking in Tad or its stimulation in Amu. Anthocyanins are well documented to accumulate upon drought and to confer desiccation tolerance (Chalker-Scott, 1999; Deeba et al., 2012; Nakabayashi et al., 2014). In affected plants, anthocyanins probably function as a barrier against photoinhibition. Considering such facts, one would expect higher activity in Tad. Nevertheless, results of some papers suggest that Tad is equipped by different mechanisms which enable it to prevent photoinhibition (Tardy et al., 1998; Havaux and Tardy, 1999). That could be a reason why Tad does not accumulate transcripts which code for anthocyanin-related proteins.

Recent studies reported an inhibition of root growth by aldehyde dehydrogenase RF2C in rice plants (Chi et al., 2013), which expression was reduced in Tad crowns relative to Amu in our study. Therefore we suggest that Tad might either inhibits or does not accumulate RF2C in order to sustain root growth.

## Conclusion

Both genotypes revealed tissue dehydration under drought conditions as shown at WSD and OP data; however, dehydration was more severe in Amu than in Tad under the same ambient conditions. It can be proposed that Tad adopts better water-saving strategy under drought stress.

Both genotypes induced several genes coded for stress-responsive signaling proteins (protein kinase HvPKABA1, protein phosphatase 2C-like protein), transcription factors as well as effector genes encoding proteins directly involved in stress acclimation - ROS scavenging enzymes (peroxidase), Cor/Lea genes (dehydrin DHN2-10, 12; LEA protein, cold acclimation protein WCOR413, proline rich protein homolog WCOR518), cold shock proteins, chaperones from HSP family (HSP70), PDI-like protein (protein disulfide isomerase catalyzing formation of disulfide bonds – role in redox homeostasis and protein folding – conformation), lipid transfer proteins, enzymes involved in a biosynthesis of stress-protective metabolites and osmolytes such as proline (delta1-pyrroline-5-carboxylate synthetase – a key enzyme in proline biosynthesis), and others.

Amu revealed under drought stress conditions a higher level of transcripts associated with processes involved in an active plant growth and development – PRR factor (psudo-response regulator – a part of a regulatory pathway leading via CONSTANS and VRN3/FT1 genes to induction of vegetative-to-reproductive phase transition under long-day conditions, photosynthesis-related proteins (chlorophyll a/b-binding proteins), cell wall elongation (expansin, cellulase, cellulose synthase), biosynthesis of growth-inducing phytohormones such as gibberellins (ent-kaurene synthase). As a consequence of higher growth rate, Amu reveals a more severe dehydration (higher WSD and lower OP values) than Tad.

Tad reveals a conservative, water-saving strategy (lower WSD, higher ABA) including a cessation of plant growth and an enhanced cell wall lignification as indicated by an enhanced biosynthesis of phenolic compounds (aromatic compounds – shikimate kinase, chloroplast precursor; lignin – caffeic acid *O*-methyltransferase) as well as other compounds with protective functions.

Several dehydrin genes including high-molecular Kn type dehydrin 5 (DHN5) as well as low-molecular YxSKn type dehydrins found in immunoblots (protein gel blots) are induced by ABA. Although Tad revealed higher levels of ABA, dehydrin protein accumulation was lower in Tad than in Amu. Regarding dehydrin transcripts, all drought-treated samples (Amu and Tad leaves and crowns) revealed enhanced levels of dehydrin transcripts with respect to control samples.

In leaf Tad effectively stimulates expression of genes encoding proteins and enzymes involved in protein folding [molecular chaperones (HSPs)], sulfur metabolism (APR, CSase, HMT-3), ROS detoxification (PAP, ferritin), Lipid biosynthesis or transport (GDSL, LTP), signal transduction (receptor protein kinase, FKPB77) or amino acid biosynthesis (GAD and anthranilate-synthase alpha 1 subunit).

In crowns, genes encoding proteins and enzymes involved in cell wall lignification (COMT, shikimate kinase), ABRE-dependent ABA signaling (ABF2), nucleosome remodeling (histone H3, H4) were strongly stimulated in Tad along with genes for numerous jasmonate induced proteins (OPR, thionin, JIP23) suggesting the important role of crowns in water deprivation response.

## Author Contributions

All authors contributed equally to this work. PS and VS have done bioinformatics analysis, data comparison and data evaluation and wrote the text. JO and AJ suggested the study design, participated in data evaluation and wrote the text. KK, PV, and IP conducted the water-relationship parameters measurement, evaluated the data and wrote the text. All authors read and approved the final manuscript.

## Conflict of Interest Statement

The authors declare that the research was conducted in the absence of any commercial or financial relationships that could be construed as a potential conflict of interest.

## Supplementary Material

FIGURE S1 **Pricnipal component analysis of DEGs**.
