# Supplementary figures and images for: Global Scale Transcriptional Profiling of Two Contrasting Barley Genotypes Exposed to Moderate Drought Conditions: Contribution of Leaves and Crowns to Water Shortage Coping Strategies
Source: Front Plant Sci. 2016 Dec 27;7:1958. doi: 10.3389/fpls.2016.01958 (PMC5187378; doi:10.3389/fpls.2016.01958)

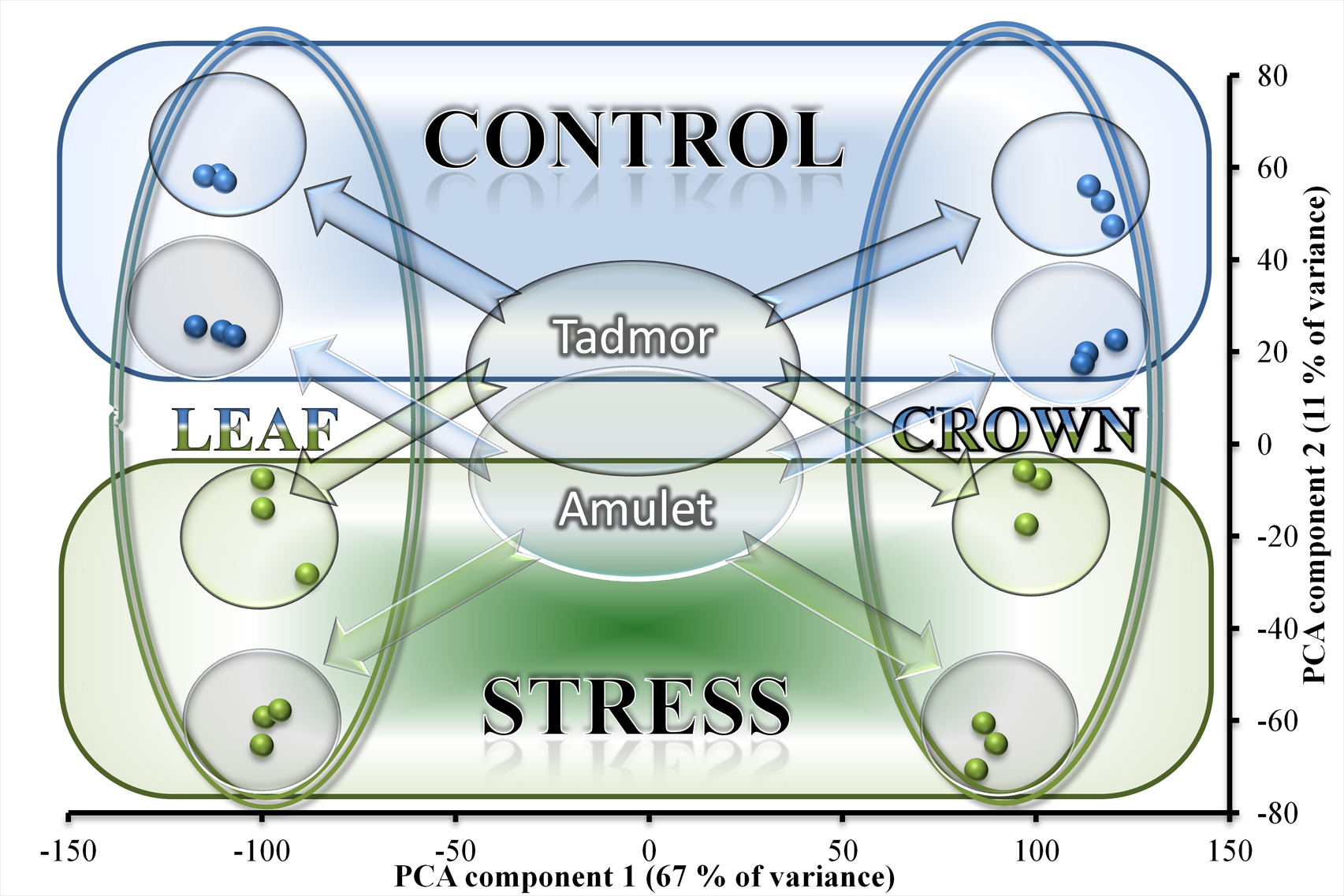

Supplement: FIGURE S1 — Pricnipal component analysis of DEGs. [file Image_1.TIF]
