# Supplementary material for: Global Scale Transcriptional Profiling of Two Contrasting Barley Genotypes Exposed to Moderate Drought Conditions: Contribution of Leaves and Crowns to Water Shortage Coping Strategies
Source: Front Plant Sci. 2016 Dec 27;7:1958. doi: 10.3389/fpls.2016.01958 (PMC5187378; doi:10.3389/fpls.2016.01958)
Supplement: Supplementary file 2 [file Table_1.DOCX]

## Supplementary Table 1

## Differentially expressed genes common to all treated samples

| ID^a^ | Log2 Fold Change^b^ | | | | Affymetrix kompletní anotace^c^ | AGI^d^ |
| --- | --- | --- | --- | --- | --- | --- |
|  | Amu  leaf | Amu  crown | Tad  leaf | Tad  crown |  |  |
| Contig1724_s_at | 8.08 | 7.59 | 8.09 | 6.98 | BEST BLASTX NR: 11/07/02 AAD02255.1 6e-19 (AF043089) dehydrin 3 [Hordeum vulgare] [Hordeum vulgare subsp. vulgare] | AT5G66400.1 |
| Contig1713_s_at | 7.85 | 8.43 | 7.69 | 7.07 | BEST BLASTX NR: 10/13/02 AAF01692.1 2e-05 (AF181454) dehydrin; DHN4 [Hordeum vulgare] [Hordeum vulgare subsp. vulgare] | AT5G66400.1 |
| Contig3814_at | 6.52 | 3.40 | 6.30 | 2.01 | BEST BLASTX NR: 11/08/02 BAB64280.1 e-122 putative delta l pyrroline-5-carboxylate synthetase [Oryza sativa (japonica cultivar-group)] | AT2G39800.1 |
| Contig1709_at | 7.49 | 7.55 | 7.31 | 6.53 | BEST BLASTX NR: 11/07/02 AAD02258.1 5e-28 (AF043092) dehydrin 7 [Hordeum vulgare] [Hordeum vulgare subsp. vulgare] | AT5G66400.1 |
| Contig15682_at | 7.98 | 8.44 | 6.94 | 6.75 | BEST BLASTX NR: <none> | AT5G36680.1 |
| HVSMEi0008A06f2_s_at | 5.35 | 6.03 | 4.50 | 4.96 | BEST BLASTX NR: 10/02/02 BAB90450.1 8e-21 (AP003709) hypothetical protein [Oryza sativa (japonica cultivar-group)] | AT2G21820.1 |
| S0000200065C10F1_at | 6.37 | 6.82 | 5.78 | 5.78 | BEST BLASTX NR: <none> |  |
| Contig7433_at | 4.62 | 2.99 | 3.95 | 2.17 | BEST BLASTX NR: 11/06/02 BAB61735.1 e-122 (AB058923) protein kinase HvPKABA1 [Hordeum vulgare subsp. vulgare] dbj\|BAB61736.1\| (AB058924) protein kinase HvPKABA1 [Hordeum vulgare subs | AT4G33950.1 |
| Dhn10(Morex)_s_at | 6.47 | 6.99 | 4.21 | 3.79 | BEST BLASTX NR: 11/07/02 AAF01698.1 1e-59 (AF181460) dehydrin; DHN10 [Hordeum vulgare] [Hordeum vulgare subsp. vulgare] | AT3G50970.1 |
| Contig7433_s_at | 4.60 | 2.95 | 4.09 | 2.40 | BEST BLASTX NR: 11/06/02 BAB61735.1 e-122 (AB058923) protein kinase HvPKABA1 [Hordeum vulgare subsp. vulgare] dbj\|BAB61736.1\| (AB058924) protein kinase HvPKABA1 [Hordeum vulgare subs | AT4G33950.1 |
| Contig10726_at | 5.18 | 4.71 | 5.72 | 4.14 | BEST BLASTX NR: 11/06/02 AAC72195.1 e-100 pyruvate dehydrogenase E1 alpha subunit [Zea mays] | AT1G24180.1 |
| Contig21426_at | 6.25 | 3.89 | 4.86 | 3.50 | BEST BLASTX NR: 11/06/02 NP_201140.1 5e-06 (NM_125730) putative protein; protein id: At5g63350.1 [Arabidopsis thaliana] | AT5G63350.1 |
| HVSMEa0014K13r2_s_at | -3.89 | -2.59 | -3.07 | -2.20 | BEST BLASTX NR: 11/08/02 NP_194529.1 3e-19 (NM_118938) putative protein; protein id: At4g28000.1 [Arabidopsis thaliana] | AT5G52882.1 |
| Contig2737_at | 5.94 | 2.76 | 5.25 | 3.04 | BEST BLASTX NR: 10/02/02 AAK07429.1 2e-80 beta-glucosidase [Musa acuminata] | AT1G02850.2 |
| HU10K22u_s_at | -4.45 | -3.99 | -3.61 | -2.36 | BEST BLASTX NR: 11/04/02 AAM08620.1 5e-44 Putative Glucan 1,3-beta-glucosidase precursor [Oryza sativa (japonica cultivar-group)] | AT2G18470.1 |
| Contig1718_s_at | 5.16 | 5.32 | 4.02 | 4.25 | BEST BLASTX NR: 11/04/02 AAD02260.1 3e-30 (AF043094) dehydrin 9 [Hordeum vulgare] [Hordeum vulgare subsp. vulgare] | AT3G50980.1 |
| Contig4281_at | 3.55 | 4.48 | 2.83 | 3.42 | BEST BLASTX NR: 11/06/02 CAC12881.1 2e-77 cold-regulated protein [Hordeum vulgare subsp. vulgare] | AT5G01300.1 |
| Contig12748_at | 5.75 | 5.10 | 4.32 | 3.71 | BEST BLASTX NR: <none> |  |
| EBem10_SQ001_I24_s_at | 5.43 | 6.31 | 3.85 | 5.14 | BEST BLASTX NR: <none> | AT1G16770.2 |
| HV10J12u_s_at | 4.39 | 5.37 | 5.39 | 3.56 | BEST BLASTX NR: 11/04/02 AAG28460.1 8e-11 actin depolymerization factor-like protein [Lophopyrum elongatum] gb\|AAG28490.1\|AF196350_1 actin depolymerization factor-like protein [Lopho | AT5G59880.1 |
| Contig13855_at | 3.92 | 5.01 | 3.81 | 4.14 | BEST BLASTX NR: 11/06/02 AAN05568.1 1e-29 hypothetical protein [Oryza sativa (japonica cultivar-group)] | AT3G07030.1 |
| Contig7112_at | 4.59 | 5.62 | 3.14 | 4.83 | BEST BLASTX NR: 10/27/02 NP_201479.1 9e-19 putative protein; protein id: At5g66780.1, supported by cDNA: gi_15081693, supported by cDNA: gi_18252266 [Arabidopsis | AT5G66780.1 |
| Contig21613_at | 5.06 | 3.23 | 3.81 | 2.37 | BEST BLASTX NR: 11/07/02 BAB89059.1 7e-35 (AP003290) PDI-like protein [Oryza sativa (japonica cultivar-group)] | AT1G60420.1 |
| Contig4991_s_at | 3.46 | 2.97 | 2.83 | 2.41 | BEST BLASTX NR: 10/02/02 AAD30232.1 3e-28 (AC007202) Is a member of the PF\|00171 aldehyde dehydrogenase family. ESTs gb\|T21534, gb\|N65241 and gb\|AA395614 come | AT1G79440.1 |
| Contig13089_at | 4.25 | 5.28 | 3.51 | 3.91 | BEST BLASTX NR: 10/26/02 AAF24564.1 e-100 (AC007764) F22C12.12 [Arabidopsis thaliana] | AT1G64110.3 |
| Contig22873_at | 4.82 | 4.28 | 4.86 | 3.45 | BEST BLASTX NR: <none> | AT4G13340.1 |
| Contig4281_s_at | 4.68 | 4.64 | 2.68 | 4.02 | BEST BLASTX NR: 11/06/02 CAC12881.1 2e-77 cold-regulated protein [Hordeum vulgare subsp. vulgare] | AT5G01300.1 |
| Contig18582_at | 3.87 | 4.46 | 3.09 | 3.55 | BEST BLASTX NR: 11/06/02 AAD17804.1 5e-45 nodule-enhanced protein phosphatase type 2C [Lotus japonicus] | AT3G11410.1 |
| Contig13161_at | 3.53 | 3.40 | 2.73 | 3.25 | BEST BLASTX NR: 10/27/02 BAC05575.1 4e-69 protein phosphatase 2C-like protein [Oryza sativa (japonica cultivar-group)] | AT1G72770.3 |
| Contig3112_at | 4.11 | 4.13 | 2.01 | 3.60 | BEST BLASTX NR: 10/13/02 T06810 2e-87 cold acclimation protein WCOR413 - wheat gb\|AAB18207.1\| cold acclimation protein WCOR413 [Triticum aestivum] | AT2G15970.1 |
| Contig11696_at | 5.73 | 5.12 | 6.53 | 4.68 | BEST BLASTX NR: 10/02/02 BAB89728.1 5e-66 putative peptide chain release factor subunit 1 (ERF1) [Oryza sativa (japonica cultivar-group)] | AT3G26618.1 |
| HVSMEl0016K12r2_s_at | -2.67 | -3.41 | -2.45 | -2.58 | BEST BLASTX NR: <none> | AT1G19600.1 |
| Contig3594_at | 5.17 | 5.27 | 3.95 | 4.56 | BEST BLASTX NR: 11/06/02 AAD02495.1 5e-91 unknown [Oryza sativa] gb\|AAD02496.1\| embryo-specific protein [Oryza sativa subsp. indica] | AT1G05510.1 |
| Contig1678_s_at | -5.06 | -2.79 | -3.73 | -2.03 | BEST BLASTX NR: 11/06/02 P32024 e-103 23 KD JASMONATE-INDUCED PROTEIN pir\|\|S22514 jasmonate-induced protein 1 - barley |  |
| Contig1725_s_at | 7.37 | 9.02 | 7.30 | 7.17 | BEST BLASTX NR: 11/06/02 P12951 3e-29 DEHYDRIN DHN1 (B8) pir\|\|S05544 dehydrin 8 - barley | AT5G66400.1 |
| Contig13753_at | 5.34 | 6.50 | 3.50 | 2.96 | BEST BLASTX NR: 11/07/02 AAD02261.1 6e-64 (AF043095) dehydrin 10 [Hordeum vulgare] [Hordeum vulgare subsp. vulgare] | AT2G21490.1 |
| Contig20976_at | 3.25 | 3.44 | 2.08 | 2.84 | BEST BLASTX NR: 10/13/02 T02084 2e-22 G-box binding factor 1 - maize gb\|AAA80169.1\| G-box binding factor 1 | AT2G46270.1 |
| Contig10934_at | 5.09 | 6.29 | 3.22 | 5.28 | BEST BLASTX NR: 11/08/02 AAN06848.1 2e-16 (AC099401) Putative abscisic acid-induced protein [Oryza sativa (japonica cultivar-group)] | AT3G22490.1 |
| Contig44_at | 4.17 | 6.85 | 3.48 | 3.69 | BEST BLASTX NR: 11/04/02 NP_187864.1 6e-35 heat shock protein 70; protein id: At3g12580.1 [Arabidopsis thaliana] | AT3G12580.1 |
| HVSMEi0006K11r2_s_at | 4.76 | 5.47 | 4.12 | 4.31 | BEST BLASTX NR: 10/02/02 BAB91939.1 1e-32 (AP003768) putative NADP dependent malic enzyme [Oryza sativa (japonica cultivar-group)] | AT2G19900.1 |
| Contig17685_at | 3.69 | 5.59 | 3.02 | 3.44 | BEST BLASTX NR: 10/27/02 NP_174120.1 1e-59 (NM_102564) unknown protein; protein id: At1g27990.1 [Arabidopsis thaliana] | AT1G27990.1 |
| Contig8085_at | 3.37 | 3.33 | 4.50 | 3.04 | BEST BLASTX NR: 11/08/02 T10443 6e-55 probable major protein body membrane protein MP27 / major protein body protein MP32 precursor - cucurbit |  |
| Contig1701_s_at | 6.19 | 7.44 | 6.83 | 5.99 | BEST BLASTX NR: 11/07/02 AAD02254.1 1e-30 (AF043088) dehydrin 2 [Hordeum vulgare] [Hordeum vulgare subsp. vulgare] | AT3G50980.1 |
| Contig8824_at | 4.32 | 5.53 | 3.31 | 4.78 | BEST BLASTX NR: 11/04/02 NP_194911.1 4e-18 (NM_119333) putative protein; protein id: At4g31830.1 [Arabidopsis thaliana] | AT4G31830.1 |
| Contig12746_at | 3.30 | 4.94 | 2.92 | 4.25 | BEST BLASTX NR: <none> | AT2G29210.1 |
| HU03I15u_s_at | 2.73 | 3.29 | 2.63 | 2.96 | BEST BLASTX NR: <none> | AT3G11410.1 |
| Contig2408_at | 5.49 | 4.58 | 5.19 | 3.91 | BEST BLASTX NR: 10/26/02 T04147 2e-33 LEA protein - rice gb\|AAC03364.1\| LEA-like protein [Oryza sativa] | AT3G15670.1 |
| Contig24328_at | -4.49 | -3.13 | -2.05 | -2.20 | BEST BLASTX NR: 11/08/02 BAC16424.1 6e-22 P0045F02.11 [Oryza sativa (japonica cultivar-group)] | AT5G48485.1 |
| Contig8895_at | -2.32 | -3.27 | -2.39 | -2.65 | BEST BLASTX NR: 01/29/03 BAB16906.1 e-104 P0005A05.10 [Oryza sativa (japonica cultivar-group)] | AT1G19600.1 |
| Contig8058_at | 5.54 | 5.41 | 3.42 | 4.08 | BEST BLASTX NR: 11/04/02 Q00747 7e-07 PROTEIN LE25 pir\|\|S19253 gene le25 protein - tomato | AT5G06760.1 |
| Contig1721_at | 5.22 | 7.55 | 6.16 | 6.13 | BEST BLASTX NR: 11/07/02 AAF01690.1 6e-37 (AF181452) dehydrin; Dhn2 [Hordeum vulgare] [Hordeum vulgare subsp. vulgare] | AT5G66400.1 |
| Contig9382_at | 3.95 | 5.11 | 3.07 | 4.26 | BEST BLASTX NR: <none> | AT1G16610.2 |
| Contig2407_s_at | 6.99 | 6.60 | 6.23 | 5.08 | BEST BLASTX NR: 11/06/02 P14928 5e-66 ABA-inducible protein PHV A1 pir\|\|S08313 abscisic acid-induced protein HVA-1 - barley | AT3G15670.1 |
| Contig9143_at | 3.48 | 4.67 | 3.98 | 4.00 | BEST BLASTX NR: <none> | AT3G22142.1 |
| Contig5481_at | 3.06 | 5.82 | 4.77 | 4.16 | BEST BLASTX NR: 10/28/02 NP_651785.1 4e-007 (NM_143528) CG9682 gene product [Drosophila melanogaster] gb\|AAL29083.1\| (AY061535) LP01629p [Drosophila melanogaster] | AT2G42560.1 |
| rbah13p07_s_at | -5.83 | -5.50 | -3.50 | -2.89 | BEST BLASTX NR: 10/02/02 AAM76682.1 2e-24 (AF387866) peroxidase [Triticum aestivum] | AT5G05340.1 |
| Contig5807_s_at | -3.56 | -4.25 | -2.89 | -2.32 | BEST BLASTX NR: <none> |  |
| Contig16787_at | 2.37 | 2.38 | 2.09 | 2.09 | BEST BLASTX NR: 10/29/02 NP_192874.1 5e-04 (NM_117206) putative protein; protein id: At4g11350.1 [Arabidopsis thaliana] |  |
| Contig3811_at | 3.26 | 3.43 | 4.40 | 2.89 | BEST BLASTX NR: 10/13/02 T07610 1e-95 WSI76 protein - rice dbj\|BAA05538.1\| WSI76 protein induced by water stress [Oryza sativa] | AT2G47180.1 |
| Contig19400_at | 2.81 | 3.77 | 3.36 | 2.03 | BEST BLASTX NR: <none> |  |
| Contig19992_at | 3.01 | 4.75 | 2.77 | 3.92 | BEST BLASTX NR: 10/27/02 T06199 2e-24 probable lipid transfer protein - barley gb\|AAB47967.1\| nonspecific lipid transfer protein [Hordeum vulgare] | AT3G18280.1 |
| Contig8708_at | 2.59 | 5.14 | 2.15 | 5.32 | BEST BLASTX NR: 10/26/02 CAA69976.1 2e-64 MtN3 [Medicago truncatula] | AT5G13170.1 |
| Contig15430_at | 2.68 | 4.19 | 3.71 | 3.44 | BEST BLASTX NR: 10/29/02 Q42908 e-102 2,3-bisphosphoglycerate-independent phosphoglycerate mutase (Phosphoglyceromutase) (BPG-independent PGAM) (PGAM-I) | AT1G09780.1 |
| Contig5994_s_at | 3.67 | 2.40 | 3.66 | 2.38 | BEST BLASTX NR: 10/13/02 P22220 7e-81 ARGININE DECARBOXYLASE (ARGDC) (ADC) pir\|\|S12265 arginine decarboxylase (EC 4.1.1.19) - oat | AT4G34710.2 |
| Contig14416_at | 2.32 | 5.15 | 2.46 | 4.65 | BEST BLASTX NR: 10/13/02 NP_566365.1 2e-24 expressed protein; protein id: At3g10020.1, supported by cDNA: 7073., supported by cDNA: gi_15081659, supported by | AT3G10020.1 |
| Contig19500_at | 3.28 | 6.57 | 3.79 | 5.04 | BEST BLASTX NR: <none> |  |
| Contig3807_at | 3.93 | 2.76 | 4.36 | 3.58 | BEST BLASTX NR: 10/28/02 BAB91740.1 1e-061 putative nifU-like protein [Oryza sativa (japonica cultivar-group)] | AT4G22220.1 |
| Contig5454_at | 3.23 | 6.27 | 3.72 | 5.62 | BEST BLASTX NR: 10/27/02 AAL23749.1 7e-66 (AY057933) stress-inducible membrane pore protein [Bromus inermis] | AT4G16160.2 |
| Contig4621_at | -3.81 | -3.12 | -4.32 | -2.49 | BEST BLASTX NR: 10/13/02 T06806 5e-50 proline rich protein homolog WCOR518 - wheat (fragment) gb\|AAB18205.1\| cold acclimation protein WCOR518 [Triticum aestivum] | AT3G22120.1 |
| Contig3426_at | 2.77 | 3.79 | 3.24 | 2.17 | BEST BLASTX NR: 10/29/02 P14895 5e-50 High molecular mass early light-inducible protein HV58, chloroplast precursor (ELIP) | AT3G22840.1 |
| Contig44_s_at | 2.46 | 3.95 | 3.37 | 2.76 | BEST BLASTX NR: 11/04/02 NP_187864.1 6e-35 heat shock protein 70; protein id: At3g12580.1 [Arabidopsis thaliana] | AT3G12580.1 |
| Contig10206_s_at | 2.35 | 4.29 | 3.35 | 3.44 | BEST BLASTX NR: <none> | AT1G20130.2 |
| Contig6087_s_at | 3.70 | 7.92 | 3.47 | 6.13 | BEST BLASTX NR: 10/02/02 NP_196350.1 1e-21 putative protein; protein id: At5g07330.1 [Arabidopsis thaliana] pir\|\|T49872 hypothetical protein T2I1.40 - Arabidopsis thaliana | AT5G07330.1 |
| Contig2546_at | 2.30 | 2.36 | 2.37 | 2.12 | BEST BLASTX NR: 10/29/02 T06486 3e-64 barwin homolog wheatwin2 precursor - wheat emb\|CAA06857.1\| (AJ006099) wheatwin2 [Triticum aestivum] | AT3G04720.1 |
| Contig13676_at | -2.42 | -3.08 | -2.01 | -2.02 | BEST BLASTX NR: 11/06/02 BAB64202.1 3e-32 (AP003266) putative glutaredoxin [Oryza sativa (japonica cultivar-group)] | AT4G15700.1 |
| Contig4622_s_at | -2.84 | -3.61 | -2.86 | -2.07 | BEST BLASTX NR: 11/08/02 T06806 4e-26 proline rich protein homolog WCOR518 - wheat (fragment) gb\|AAB18205.1\| cold acclimation protein WCOR518 [Triticum aestivum] | AT3G22142.1 |
| Contig13674_at | -2.40 | -3.83 | -3.10 | -2.34 | BEST BLASTX NR: 11/04/02 AAK38482.1 1e-80 beta-D-xylosidase [Hordeum vulgare] | AT1G78060.1 |
| HV_CEb0001H12r2_at | -2.16 | -4.08 | -2.02 | -2.75 | BEST BLASTX NR: 11/04/02 BAC06204.1 4e-37 (AP003227) putative amino acid permease 6 [Oryza sativa (japonica cultivar-group)] | AT1G58360.1 |
| Contig7315_at | 2.21 | -3.47 | 2.04 | -2.00 | BEST BLASTX NR: <none> |  |
| HVSMEm0005P05r2_at | -3.83 | -6.62 | -3.59 | -4.00 | BEST BLASTX NR: 11/04/02 S14611 2e-23 peroxidase (EC 1.11.1.7) - barley (fragment) | AT5G05340.1 |

^a^ Affymetrix 22 K Barley1 GeneChip Genome Array probe ID

^b^ Log2 transformed expression difference of treated samples against parallel controls

^c^ Microarray manufacturer (Affymetrix) annotation of individual IDs

^d^*Arabidopsis* locus identifier corresponding to individual IDs
