# Supplementary material for: Global Scale Transcriptional Profiling of Two Contrasting Barley Genotypes Exposed to Moderate Drought Conditions: Contribution of Leaves and Crowns to Water Shortage Coping Strategies
Source: Front Plant Sci. 2016 Dec 27;7:1958. doi: 10.3389/fpls.2016.01958 (PMC5187378; doi:10.3389/fpls.2016.01958)
Supplement: Supplementary file 3 [file Table_2.DOCX]

### Supplementary Table 2

### Genes whose expression is enhanced in Tad relative to Amu in leaf

| ID^a^ | Tad x Amu Log2 FC^b^ | | Affymetrix annotation^c^ | AGI^d^ |
| --- | --- | --- | --- | --- |
|  | **crown** | **leaf** |  |  |
| Contig4027_at | 0.191 | 2.31 | BEST BLASTX NR: 11/07/02 AAL66290.1 3e-67 adenosine 5'-phosphosulfate reductase [Glycine max] | AT4G04610.1 |
| Contig9567_at | -1.318 | 2.244 | BEST BLASTX NR: 10/27/02 CAC27140.1 6e-97 ADP, ATP carrier protein precursor [Picea abies] | AT4G28390.1 |
| Contig3858_at | 1.449 | 2.198 | BEST BLASTX NR: 10/29/02 P23901 e-140 Aldose reductase (AR) (Aldehyde reductase) pir\|\|S15024 aldose reductase-related protein - barley | AT5G01670.1 |
| Contig11179_at | 0.458 | 2.25 | BEST BLASTX NR: 10/27/02 AAK82452.1 e-127 (AC091247) anthranilate synthase alpha 1 subunit [Oryza sativa] gb\|AAL79757.1\|AC096687_21 (AC096687) anthranilate synthase alpha 1 subunit [ | AT3G55870.1 |
| Contig3332_at | 0.55 | 2.268 | BEST BLASTX NR: 10/04/02 AAG32960.1 4e-55 apyrase GS52 [Glycine soja] | AT5G18280.1 |
| Contig16980_at | -0.575 | 3.151 | BEST BLASTX NR: 11/06/02 NP_567058.1 2e-49 auxin-regulated protein; protein id: At3g57810.1, supported by cDNA: 101256., supported by cDNA: 29384. [Arabidopsis | AT3G57810.3 |
| HV09F14u_at | -0.007 | 2.194 | BEST BLASTX NR: 11/04/02 AAF71261.2 4e-05 (AF232008) beta-glucosidase aggregating factor precursor [Zea mays] | AT3G16460.2 |
| Contig5737_s_at | 0.727 | 2.226 | BEST BLASTX NR: 11/08/02 CAB53482.1 1e-80 CAA30379.1 protein [Oryza sativa] | AT1G45688.1 |
| Contig1337_s_at | 0.648 | 2.354 | BEST BLASTX NR: 11/06/02 P23252 6e-48 COLD-REGULATED PROTEIN 2 pir\|\|B45512 cold-regulated protein 2 - barley (fragment) | AT5G42540.1 |
| Contig9959_at | 0.177 | 2.745 | BEST BLASTX NR: 11/08/02 BAC10135.1 6e-34 (AP004339) contains EST C29177(C63963)~similar to zinc finger protein [Oryza sativa (japonica cultivar-group)] | AT5G64920.1 |
| Contig1404_at | 1.93 | 2.824 | BEST BLASTX NR: 11/08/02 BAC20892.1 1e-83 contains ESTs AU172828(E31314),C74435(E31314)~similar to nodulin [Oryza sativa (japonica cultivar-group)] | AT3G26430.1 |
| Contig1404_x_at | 1.409 | 2.748 | BEST BLASTX NR: 11/08/02 BAC20892.1 1e-83 contains ESTs AU172828(E31314),C74435(E31314)~similar to nodulin [Oryza sativa (japonica cultivar-group)] | AT3G26430.1 |
| HV12E23u_at | 0.422 | 2.715 | BEST BLASTX NR: 11/04/02 AAD23908.1 1e-45 (AF073696) cysteine synthase [Oryza sativa] [Oryza sativa (japonica cultivar-group)] | AT3G22460.1 |
| Contig3217_s_at | 1.329 | 2.901 | BEST BLASTX NR: 10/29/02 BAC10287.1 9e-24 defensin [Triticum aestivum] | AT2G02100.1 |
| Contig632_s_at | 0.726 | 2.094 | BEST BLASTX NR: 11/04/02 P42824 e-100 DnaJ protein homolog 2 pir\|\|S42031 LDJ2 protein - leek | AT3G44110.1 |
| Contig11487_at | 1.074 | 2.007 | BEST BLASTX NR: 11/06/02 AAM63624.1 2e-33 DnaJ protein, putative [Arabidopsis thaliana] | AT1G56300.1 |
| Contig5448_at | 0.488 | 2.373 | BEST BLASTX NR: 10/27/02 AAF75090.1 2e-42 (AC007583) ESTs gb\|Z27026 and gb\|29860 come from this gene. [Arabidopsis thaliana] | AT1G07645.1 |
| Contig18451_at | -1.122 | 2.295 | BEST BLASTX NR: 11/04/02 BAA92727.1 2e-04 ESTs AU082409(E30736),C74252(E30736) correspond to a region of the predicted gene.~hypothetical protein [Oryza sativa |  |
| Contig5345_s_at | -0.003 | 3.202 | BEST BLASTX NR: 11/04/02 NP_566623.1 9e-71 expressed protein; protein id: At3g19000.1, supported by cDNA: 36985., supported by cDNA: gi_20466683 [Arabidopsis | AT3G19000.1 |
| Contig5345_at | 0.523 | 2.285 | BEST BLASTX NR: 11/04/02 NP_566623.1 9e-71 expressed protein; protein id: At3g19000.1, supported by cDNA: 36985., supported by cDNA: gi_20466683 [Arabidopsis | AT3G19000.1 |
| Contig2714_at | -0.686 | 2.083 | BEST BLASTX NR: 11/07/02 P29036 3e-71 Ferritin 1, chloroplast precursor pir\|\|S22498 ferritin 1 precursor (clone FM1) - maize (fragment) | AT3G56090.1 |
| Contig11789_at | 0.144 | 2.087 | BEST BLASTX NR: 11/06/02 T04363 5e-58 FIERG2 protein - rice gb\|AAC04628.1\| Os-FIERG2 gene product [Oryza sativa] | AT3G55470.2 |
| Contig15_s_at | 1.779 | 2.554 | BEST BLASTX NR: 11/06/02 NP_200316.1 3e-15 GDSL-motif lipase/hydrolase-like protein; protein id: At5g55050.1, supported by cDNA: gi_18175651 [Arabidopsis thaliana] | AT5G55050.1 |
| Contig1373_at | 1.079 | 2.456 | BEST BLASTX NR: 11/08/02 NP_197235.1 e-112 glutamate decarboxylase 1 (GAD 1); protein id: At5g17330.1, supported by cDNA: gi_20453186, supported by cDNA: | AT2G02000.1 |
| HY03K23u_at | -0.613 | 3.423 | BEST BLASTX NR: 11/08/02 S72544 4e-36 heat shock protein 17.9 - pearl millet emb\|CAA63903.1\| heat shock protein 17.9 [Pennisetum glaucum] | AT5G59720.1 |
| Contig2012_s_at | -0.485 | 3.338 | BEST BLASTX NR: 11/08/02 S72544 8e-57 heat shock protein 17.9 - pearl millet emb\|CAA63903.1\| heat shock protein 17.9 [Pennisetum glaucum] | AT5G59720.1 |
| Contig998_s_at | 0.516 | 2.171 | BEST BLASTX NR: 11/08/02 CAA47948.2 2e-70 heat shock protein 70 [Oryza sativa (indica cultivar-group)] | AT3G12580.1 |
| Contig1206_s_at | 0.508 | 2.667 | BEST BLASTX NR: 11/06/02 AAD11549.1 e-123 heat shock protein 80 [Triticum aestivum] | AT5G56010.1 |
| Contig1207_at | 0.77 | 2.205 | BEST BLASTX NR: 11/06/02 AAD11549.1 5e-78 heat shock protein 80 [Triticum aestivum] | AT5G56010.1 |
| Contig10029_at | -1.629 | 2.285 | BEST BLASTX NR: 10/29/02 NP_175807.1 6e-26 heat-shock protein, putative; protein id: At1g54050.1, supported by cDNA: 97415., supported by cDNA: gi_13194789 | AT1G54050.1 |
| Contig3552_at | 0.967 | 4.862 | BEST BLASTX NR: 10/13/02 T01354 3e-51 herbicide safener binding protein 1 - maize gb\|AAC12715.1\| herbicide safener binding protein [Zea mays] | AT1G63140.2 |
| Contig6344_s_at | 0.394 | 2.073 | BEST BLASTX NR: 11/06/02 T01354 8e-58 herbicide safener binding protein 1 - maize gb\|AAC12715.1\| herbicide safener binding protein [Zea mays] | AT4G35160.1 |
| Contig7937_s_at | 1.153 | 2.558 | BEST BLASTX NR: 11/04/02 AAF76254.1 e-102 high pI alpha-glucosidase [Hordeum vulgare] | AT5G11720.1 |
| HVSMEl0014E16r2_s_at | -0.252 | 2.107 | BEST BLASTX NR: 11/04/02 AAG22539.1 3e-16 homocysteine S-methyltransferase-3 [Zea mays] | AT3G63250.1 |
| Contig3310_at | -0.149 | 2.056 | BEST BLASTX NR: 11/04/02 AAG22539.1 2e-52 homocysteine S-methyltransferase-3 [Zea mays] | AT3G63250.2 |
| Contig11778_at | -0.078 | 2.066 | BEST BLASTX NR: 10/13/02 NP_565586.1 e-111 (NM_128071) HSP100/ClpB, putative; protein id: At2g25140.1, supported by cDNA: gi_17979453 [Arabidopsis thaliana] | AT2G25140.1 |
| Contig873_s_at | -1.309 | 2.211 | BEST BLASTX NR: 10/27/02 AAB99745.1 e-115 HSP70 [Triticum aestivum] | AT3G12580.1 |
| Contig10615_at | -0.674 | 2.266 | BEST BLASTX NR: 10/26/02 AAL84285.1 6e-36 hypothetical protein [Oryza sativa (japonica cultivar-group)] | AT2G35290.1 |
| HI04F14u_at | -0.435 | 2.264 | BEST BLASTX NR: 11/06/02 BAC03353.1 1e-07 (AP004821) hypothetical protein [Oryza sativa (japonica cultivar-group)] | AT1G12480.1 |
| Contig7736_at | -1.344 | 2.167 | BEST BLASTX NR: 10/13/02 BAB62552.1 2e-51 (AP003215) hypothetical protein~similar to Arabidopsis thaliana chromosome 1, F18B13.24 [Oryza sativa (japonica | AT1G80160.1 |
| Contig20064_at | 0.639 | 2.285 | BEST BLASTX NR: 11/04/02 NP_189291.1 3e-22 integral membrane protein, putative; protein id: At3g26590.1, supported by cDNA: gi_16323120 [Arabidopsis thaliana] |  |
| HB16L13r_x_at | 0.85 | 3.024 | BEST BLASTX NR: <none> | AT5G12020.1 |
| Contig16582_at | 0.261 | 2.968 | BEST BLASTX NR: <none> |  |
| Contig21196_at | -1.07 | 2.906 | BEST BLASTX NR: <none> | AT5G35090.1 |
| Contig21281_at | 0.638 | 2.671 | BEST BLASTX NR: <none> | AT1G61080.1 |
| Contig19596_at | 0.745 | 2.657 | BEST BLASTX NR: <none> |  |
| Contig6701_s_at | 0.166 | 2.555 | BEST BLASTX NR: <none> |  |
| rbags11o22_at | -0.192 | 2.526 | BEST BLASTX NR: <none> |  |
| Contig23137_at | 0.123 | 2.522 | BEST BLASTX NR: <none> |  |
| Contig22600_at | 0.712 | 2.254 | BEST BLASTX NR: <none> |  |
| HVSMEb0014F22f_s_at | 0.947 | 2.211 | BEST BLASTX NR: <none> | AT1G64660.1 |
| EBro04_SQ004_L16a_at | -1.115 | 2.197 | BEST BLASTX NR: <none> |  |
| HVSMEi0006I22r2_at | -0.152 | 2.157 | BEST BLASTX NR: <none> |  |
| Contig18530_at | -0.244 | 2.064 | BEST BLASTX NR: <none> |  |
| HVSMEk0006G12r2_s_at | -0.697 | 2.062 | BEST BLASTX NR: <none> | AT1G66150.1 |
| Contig24328_at | 0.93 | 2.441 | BEST BLASTX NR: 11/08/02 BAC16424.1 6e-22 P0045F02.11 [Oryza sativa (japonica cultivar-group)] | AT5G48485.1 |
| Contig24832_at | 0.303 | 3.635 | BEST BLASTX NR: 11/07/02 T03836 5e-32 phosphate/phosphoenolpyruvate translocator TABPPT10 precursor, plastid - common tobacco | AT5G33320.1 |
| Contig3783_at | -0.004 | 5.919 | BEST BLASTX NR: 11/06/02 AAC31615.1 2e-17 (AF001634) physical impedance induced protein [Zea mays] | AT1G12090.1 |
| Contig2720_at | -0.045 | 4.36 | BEST BLASTX NR: 10/13/02 T06489 e-110 probable peptidylprolyl isomerase (EC 5.2.1.8) FKBP77 - wheat emb\|CAA68913.1\| peptidylprolyl isomerase [Triticum aestivum] | AT3G25230.2 |
| Contig2717_s_at | 0.051 | 4.098 | BEST BLASTX NR: 11/08/02 T06489 2e-44 probable peptidylprolyl isomerase (EC 5.2.1.8) FKBP77 - wheat emb\|CAA68913.1\| peptidylprolyl isomerase [Triticum aestivum] | AT3G25230.2 |
| Contig5388_at | 0.465 | 2.688 | BEST BLASTX NR: 10/13/02 O24650 9e-60 PROFILIN 2/4 (POLLEN ALLERGEN PHL P 11) (PHL P XI) emb\|CAA70608.1\| (Y09456) profilin 2 [Phleum pratense] | AT2G19770.1 |
| Contig13114_at | 1.01 | 2.396 | BEST BLASTX NR: 11/08/02 AAG21913.1 7e-08 (AC026815) putative cyanase [Oryza sativa] | AT3G23490.1 |
| HVSMEm0003G16r2_at | 0.287 | 3.536 | BEST BLASTX NR: 11/04/02 AAK38084.1 3e-57 putative cytochrome P450 [Lolium rigidum] | AT3G26300.1 |
| Contig7490_at | 0.132 | 2.732 | BEST BLASTX NR: 10/27/02 NP_179646.1 8e-79 putative heat shock protein; protein id: At2g20560.1, supported by cDNA: 25528., supported by cDNA: gi_15982894 | AT2G20560.1 |
| rbaal35o24_at | 0.073 | 2.528 | BEST BLASTX NR: 11/08/02 AAN06862.1 4e-38 Putative heat shock protein [Oryza sativa (japonica cultivar-group)] | AT3G22830.1 |
| Contig20832_at | 0.373 | 4.509 | BEST BLASTX NR: 11/08/02 AAM65281.1 1e-26 (AY087744) putative hydrolase [Arabidopsis thaliana] | AT2G18360.1 |
| Contig3776_s_at | 0.207 | 2.679 | BEST BLASTX NR: 10/29/02 AAM74427.1 2e-18 (AC123594) Putative lipid transfer protein [Oryza sativa (japonica cultivar-group)] | AT4G12480.1 |
| Contig18051_at | 0.954 | 3.731 | BEST BLASTX NR: 11/08/02 AAL87171.1 8e-17 (AF480496) putative myb-related protein [Oryza sativa (japonica cultivar-group)] | AT4G39250.1 |
| Contig6164_s_at | 0.293 | 2.445 | BEST BLASTX NR: 10/29/02 NP_194519.1 8e-28 putative protein; protein id: At4g27900.1, supported by cDNA: 41557., supported by cDNA: gi_17064785, supported by | AT5G53420.1 |
| Contig6165_at | 0.136 | 2.253 | BEST BLASTX NR: 11/07/02 NP_194519.1 5e-18 putative protein; protein id: At4g27900.1, supported by cDNA: 41557., supported by cDNA: gi_17064785, supported by | At4g27900.1 |
| Contig6164_at | 0.301 | 2.107 | BEST BLASTX NR: 10/29/02 NP_194519.1 8e-28 putative protein; protein id: At4g27900.1, supported by cDNA: 41557., supported by cDNA: gi_17064785, supported by | AT5G53420.1 |
| Contig4066_at | -1.237 | 2.056 | BEST BLASTX NR: 10/27/02 NP_199517.1 4e-10 putative protein; protein id: At5g47060.1 [Arabidopsis thaliana] | AT4G17670.1 |
| Contig6713_at | -0.191 | 2.025 | BEST BLASTX NR: 10/29/02 NP_190948.1 6e-78 (NM_115240) putative protein; protein id: At3g53800.1, supported by cDNA: 37834. [Arabidopsis thaliana] | At3g53800.1 |
| Contig26476_at | 0.138 | 2.405 | BEST BLASTX NR: 11/06/02 BAC07354.1 6e-20 putative purple acid phosphatase [Oryza sativa (japonica cultivar-group)] | AT1G52940.1 |
| Contig5562_s_at | -0.574 | 2.111 | BEST BLASTX NR: 10/02/02 BAB56062.1 9e-66 (AP003106) putative receptor-protein kinase [Oryza sativa (japonica cultivar-group)] | AT3G51550.1 |
| HVSMEf0019H18r2_at | 0.707 | 2.201 | BEST BLASTX NR: 11/08/02 BAB63833.1 3e-32 putative tonoplast membrane integral protein [Oryza sativa (japonica cultivar-group)] | AT4G01470.1 |
| Contig18381_at | 0.136 | 2.347 | BEST BLASTX NR: 11/08/02 BAB12027.1 2e-06 putative transposable element Tip100 protein [Oryza sativa (japonica cultivar-group)] | AT1G19260.1 |
| EBma03_SQ001_K06_x_at | 0.821 | 2.237 | BEST BLASTX NR: 10/13/02 AAD51625.1 1e-39 seed maturation protein PM37 [Glycine max] | AT3G44110.1 |
| Contig25725_at | 0.174 | 3.007 | BEST BLASTX NR: 11/06/02 BAC15912.1 2e-29 (AP003811) similar to chloroplast nucleoid DNA binding protein [Oryza sativa (japonica cultivar-group)] | AT2G03200.1 |
| Contig3286_s_at | -1.849 | 3.665 | BEST BLASTX NR: 10/26/02 AAK51797.1 2e-62 small heat shock protein HSP17.8 [Triticum aestivum] | AT5G12020.1 |
| Contig3284_x_at | 0.909 | 3.28 | BEST BLASTX NR: 10/26/02 AAK51797.1 1e-54 small heat shock protein HSP17.8 [Triticum aestivum] | AT5G12020.1 |
| Contig3289_at | -0.309 | 2.576 | BEST BLASTX NR: 11/06/02 AAK51797.1 3e-64 small heat shock protein HSP17.8 [Triticum aestivum] | AT5G12020.1 |
| Contig3288_x_at | 0.842 | 2.478 | BEST BLASTX NR: 11/04/02 AAK51797.1 2e-64 small heat shock protein HSP17.8 [Triticum aestivum] | AT5G12020.1 |
| Contig3285_at | 0.409 | 2.299 | BEST BLASTX NR: 11/08/02 AAK51797.1 3e-49 small heat shock protein HSP17.8 [Triticum aestivum] | AT5G12020.1 |
| Contig3287_x_at | -0.739 | 2.276 | BEST BLASTX NR: 11/08/02 AAK51797.1 5e-57 small heat shock protein HSP17.8 [Triticum aestivum] | AT5G12020.1 |
| Contig7425_at | -0.007 | 3.282 | BEST BLASTX NR: 10/13/02 NP_176461.1 e-110 (NM_104951) TPR-repeat protein; protein id: At1g62740.1 [Arabidopsis thaliana] | AT1G62740.1 |
| Contig18424_at | -0.183 | 2.482 | BEST BLASTX NR: 11/06/02 AAC72543.1 1e-97 (AF031609) unknown [Oryza sativa] [Oryza sativa (japonica cultivar-group)] | AT5G48850.1 |
| Contig4431_s_at | 0.712 | 2.223 | BEST BLASTX NR: 11/06/02 AAK44146.1 1e-08 (AF370331) unknown protein [Arabidopsis thaliana] gb\|AAN13152.1\| (AY142583) unknown protein [Arabidopsis thaliana] | AT3G24100.1 |
| Contig13994_s_at | -0.347 | 2.098 | BEST BLASTX NR: 11/06/02 AAG16855.1 5e-12 (AC069145) unknown protein [Oryza sativa] | AT3G07090.1 |
| rbah13p07_s_at* | 2.605 | 2.332 | BEST BLASTX NR: 10/02/02 AAM76682.1 2e-24 (AF387866) peroxidase [Triticum aestivum] | AT5G05340.1 |
| Contig2112_at* | 2.374 | 2.257 | BEST BLASTX NR: 11/07/02 S61406 e-103 peroxidase (EC 1.11.1.7) 2 precursor - wheat emb\|CAA59485.1\| (X85228) peroxidase [Triticum aestivum] | AT5G05340.1 |

* Significant difference in both crown and leaf

^a^ Affymetrix 22 K Barley1 GeneChip Genome Array probe ID

^b^ Log2 transformed expression difference of Tad against Amu in crown/leaf

^c^ Microarray manufacturer (Affymetrix) annotation of individual IDs

^d^*Arabidopsis* locus identifier corresponding to individual IDs
