# Supplementary material for: Global Scale Transcriptional Profiling of Two Contrasting Barley Genotypes Exposed to Moderate Drought Conditions: Contribution of Leaves and Crowns to Water Shortage Coping Strategies
Source: Front Plant Sci. 2016 Dec 27;7:1958. doi: 10.3389/fpls.2016.01958 (PMC5187378; doi:10.3389/fpls.2016.01958)
Supplement: Supplementary file 4 [file Table_3.DOCX]

*Supplementary Tab. 3*

*Genes whose expression is reduced in Tad relative to Amu in crown*

| ID^a^ | Tad x Amu Log2 FC^b^ | | Affymetrix annotation^c^ | AGI^d^ |
| --- | --- | --- | --- | --- |
|  | **crown** | **leaf** |  |  |
| Contig2007_s_at* | -2.677 | -2.563 | BEST BLASTX NR: 10/13/02 CAA69172.1 2e-63 17 kDa class I small heat shock protein [Hordeum vulgare subsp. vulgare] | AT5G59720.1 |
| Contig2008_s_at | -3.84 | -0.793 | BEST BLASTX NR: 11/06/02 CAA69172.1 3e-67 17 kDa class I small heat shock protein [Hordeum vulgare subsp. vulgare] | AT5G59720.1 |
| Contig3685_at* | -3.732 | -3.316 | BEST BLASTX NR: 10/26/02 T06978 3e-77 ABA-induced plasma membrane protein PM 19 - wheat gb\|AAB38504.1\| ABA induced plasma membrane protein PM 19 [Triticum aestivum] | AT1G04560.1 |
| Contig3684_at* | -6.147 | -2.1 | BEST BLASTX NR: 11/06/02 T06978 5e-74 ABA-induced plasma membrane protein PM 19 - wheat gb\|AAB38504.1\| ABA induced plasma membrane protein PM 19 [Triticum aestivum] | AT1G04560.1 |
| Contig7092_at | -2.071 | -0.331 | BEST BLASTX NR: 11/08/02 AAG28460.1 6e-66 actin depolymerization factor-like protein [Lophopyrum elongatum] gb\|AAG28490.1\|AF196350_1 actin depolymerization factor-like protein [Lopho | AT5G59880.1 |
| HU02F20u_s_at | -2.808 | 0.859 | BEST BLASTX NR: 11/06/02 AAG40371.1 2e-06 AT4g27960 [Arabidopsis thaliana] | AT2G23240.1 |
| Contig15332_at | -2.962 | -1.566 | BEST BLASTX NR: 11/07/02 AAD42895.1 7e-69 (AF159882) Cen-like protein FDR2 [Oryza sativa] [Oryza sativa (indica cultivar-group)] | AT2G27550.1 |
| Contig6382_s_at | -2.076 | -0.297 | BEST BLASTX NR: 10/02/02 AAL99608.1 e-106 cytosolic aldehyde dehydrogenase RF2C [Zea mays] | AT3G24503.1 |
| Contig6933_s_at | -2.27 | -1.464 | BEST BLASTX NR: 11/08/02 BAC10287.1 4e-25 defensin [Triticum aestivum] | AT2G02100.1 |
| Dhn10(Morex)_s_at* | -3.199 | -2.265 | BEST BLASTX NR: 11/07/02 AAF01698.1 1e-59 (AF181460) dehydrin; DHN10 [Hordeum vulgare] [Hordeum vulgare subsp. vulgare] | AT3G50970.1 |
| Contig13753_at | -3.547 | -1.844 | BEST BLASTX NR: 11/07/02 AAD02261.1 6e-64 (AF043095) dehydrin 10 [Hordeum vulgare] [Hordeum vulgare subsp. vulgare] | AT2G21490.1 |
| Contig1717_s_at | -2.117 | 0.741 | BEST BLASTX NR: 11/08/02 S27761 3e-80 dehydrin Dhn5 - barley gb\|AAA32952.1\| (M95810) dehydrin DHN5 [Hordeum vulgare subsp. vulgare] | AT3G50970.1 |
| Contig1432_at | -2.494 | -0.06 | BEST BLASTX NR: 01/29/03 P30569 1e-41 EC protein I/II (Zinc-metallothionein class II) pir\|\|S27369 metallothionein E(c) [similarity] - wheat | AT2G23240.1 |
| Contig15773_at | -3.825 | -1.432 | BEST BLASTX NR: 10/28/02 NP_565890.1 3e-006 (NM_129403) Expressed protein; protein id: At2g38465.1, supported by cDNA: 123915. [Arabidopsis thaliana] | AT4G22505.1 |
| Contig2004_s_at | -3.505 | -0.452 | BEST BLASTX NR: 11/06/02 T05740 3e-70 heat shock protein 18 - barley emb\|CAA45862.1\| 18 Kd heat shock protein [Hordeum vulgare subsp. vulgare] | AT5G59720.1 |
| EBro08_SQ011_I03_at* | -2.267 | 2.152 | BEST BLASTX NR: 11/06/02 T05740 9e-26 heat shock protein 18 - barley emb\|CAA45862.1\| 18 Kd heat shock protein [Hordeum vulgare subsp. vulgare] | AT5G59720.1 |
| Contig44_at | -3.168 | -0.691 | BEST BLASTX NR: 11/04/02 NP_187864.1 6e-35 heat shock protein 70; protein id: At3g12580.1 [Arabidopsis thaliana] | AT3G12580.1 |
| Contig5614_s_at | -2.021 | 0.011 | BEST BLASTX NR: 11/08/02 T01354 3e-23 herbicide safener binding protein 1 - maize gb\|AAC12715.1\| herbicide safener binding protein [Zea mays] | AT4G35160.1 |
| Contig9057_at | -2.153 | 0.212 | BEST BLASTX NR: 10/29/02 BAB86120.1 7e-66 (AP003437) hypothetical protein~similar to Arabidopsis thaliana, F18F4.70 [Oryza sativa (japonica cultivar-group)] | AT4G19970.1 |
| Contig3431_x_at* | -2.212 | -4.423 | BEST BLASTX NR: 11/06/02 P14897 7e-63 Low molecular mass early light-inducible protein HV90, chloroplast precursor (ELIP) | AT3G22840.1 |
| rbaal14f06_s_at | -2.019 | -1.474 | BEST BLASTX NR: 10/04/02 CAB97352.1 2e-04 (AJ249144) MADS-box protein 5 [Hordeum vulgare subsp. vulgare] | AT1G69120.1 |
| Contig8119_at | -6.564 | -5.208 | BEST BLASTX NR: <none> |  |
| Contig7004_at* | -2.719 | -2.614 | BEST BLASTX NR: <none> | AT5G17210.2 |
| Contig19843_at | -3.13 | -1.859 | BEST BLASTX NR: <none> |  |
| rbasd23b02_s_at | -3.599 | -1.437 | BEST BLASTX NR: <none> | AT4G16260.1 |
| Contig21971_at | -2.288 | -0.842 | BEST BLASTX NR: <none> | AT2G44710.1 |
| Contig24835_at | -2.171 | -0.748 | BEST BLASTX NR: <none> | AT2G29210.1 |
| HV14J05u_x_at | -3.206 | -0.48 | BEST BLASTX NR: <none> |  |
| Contig5270_x_at | -2.863 | -0.236 | BEST BLASTX NR: <none> |  |
| Contig16839_at | -2.297 | -0.154 | BEST BLASTX NR: <none> |  |
| Contig3186_at | -2.633 | -0.079 | BEST BLASTX NR: <none> |  |
| Contig14848_at | -3.438 | 0.446 | BEST BLASTX NR: <none> | AT1G20130.2 |
| HS06G05u_s_at | -2.371 | 0.991 | BEST BLASTX NR: <none> | AT2G42560.1 |
| Contig9355_s_at | -2.186 | -0.075 | BEST BLASTX NR: 10/13/02 BAB64773.1 8e-71 P0583G08.8 [Oryza sativa (japonica cultivar-group)] | AT3G17020.1 |
| Contig7389_at | -2.67 | 0.27 | BEST BLASTX NR: 11/06/02 BAB87820.1 1e-69 P450 [Triticum aestivum] | AT2G30750.1 |
| rbaal9h21_s_at* | -2.271 | -3.166 | BEST BLASTX NR: 10/02/02 Q40070 2e-33 Photosystem II 10 kDa polypeptide, chloroplast precursor pir\|\|T06173 photosystem II 10K protein precursor - barley | AT1G79040.1 |
| Contig11477_at* | -2.242 | -3.028 | BEST BLASTX NR: 11/08/02 Q40070 5e-39 Photosystem II 10 kDa polypeptide, chloroplast precursor pir\|\|T06173 photosystem II 10K protein precursor - barley | AT1G79040.1 |
| Contig1053_at | -2.122 | -0.712 | BEST BLASTX NR: 11/04/02 CAA66667.1 5e-58 polyubiquitin [Pinus sylvestris] | AT4G05320.3 |
| Contig3777_at | -2.117 | -0.18 | BEST BLASTX NR: 11/04/02 BAB16431.1 2e-14 P-rich protein Nt-SubC29 [Nicotiana tabacum] | AT1G12090.1 |
| Contig3778_x_at | -2.518 | -0.006 | BEST BLASTX NR: 11/08/02 BAB16431.1 5e-14 P-rich protein Nt-SubC29 [Nicotiana tabacum] |  |
| Contig15639_at | -2.311 | -0.584 | BEST BLASTX NR: 11/06/02 AAK31286.1 3e-58 putative hexose carrier protein [Oryza sativa] gb\|AAL79779.1\|AC079874_2 putative monosaccharide transporter [Oryza sativa] | AT3G19930.1 |
| Contig8921_at | -2.042 | -0.365 | BEST BLASTX NR: 10/13/02 AAK40309.1 2e-35 putative methyl-binding domain protein MBD108 [Zea mays] | AT5G35330.3 |
| Contig12321_at | -2.42 | -1.376 | BEST BLASTX NR: 11/07/02 NP_195822.1 3e-13 putative protein; protein id: At5g02020.1, supported by cDNA: gi_16648747, supported by cDNA: gi_20334905 [Arabidopsis | AT5G02020.1 |
| Contig9398_s_at | -2.718 | -0.521 | BEST BLASTX NR: 11/08/02 NP_569030.1 9e-17 senescence-associated protein; protein id: At5g66170.1, supported by cDNA: 24140., supported by cDNA: gi_18086477, | AT5G66170.3 |
| Contig5269_x_at | -2.51 | -0.111 | BEST BLASTX NR: 11/06/02 NP_181073.1 4e-04 (NM_129082) similar to late embryogenesis abundant proteins; protein id: At2g35300.1, supported by cDNA: gi_18252896 | AT2G35300.1 |
| Contig5269_s_at | -3.588 | 0.082 | BEST BLASTX NR: 11/06/02 NP_181073.1 4e-04 (NM_129082) similar to late embryogenesis abundant proteins; protein id: At2g35300.1, supported by cDNA: gi_18252896 | AT2G35300.1 |
| Contig823_at | -2.816 | -1.459 | BEST BLASTX NR: 10/13/02 P31923 e-139 Sucrose synthase 2 (Sucrose-UDP glucosyltransferase 2) pir\|\|S32451 sucrose synthase (EC 2.4.1.13) Ss2 - barley | AT3G43190.1 |
| Contig17685_at | -2.144 | -0.671 | BEST BLASTX NR: 10/27/02 NP_174120.1 1e-59 (NM_102564) unknown protein; protein id: At1g27990.1 [Arabidopsis thaliana] | AT1G27990.1 |
| Contig4887_s_at | -2.024 | 0.543 | BEST BLASTX NR: 10/13/02 P12412 7e-21 Vignain precursor (Bean endopeptidase) (Cysteine proteinase) (Sulfhydryl-endopeptidase) (SH-EP) | AT2G34080.1 |
| Contig26042_at* | -2.075 | -2.84 | BEST BLASTX NR: 11/08/02 CAD24007.1 .068 WIRE protein [Homo sapiens] |  |
| Contig3810_at | -2.007 | 0.621 | BEST BLASTX NR: 10/04/02 T07610 5e-94 WSI76 protein - rice dbj\|BAA05538.1\| WSI76 protein induced by water stress [Oryza sativa] | AT1G09350.1 |

* Significant difference in both crown and leaf

^a^ Affymetrix 22 K Barley1 GeneChip Genome Array probe ID

^b^ Log2 transformed expression difference of Tad against Amu in crown/leaf

^c^ Microarray manufacturer (Affymetrix) annotation of individual IDs

^d^*Arabidopsis* locus identifier corresponding to individual IDs
