# Supplementary material for: Global Scale Transcriptional Profiling of Two Contrasting Barley Genotypes Exposed to Moderate Drought Conditions: Contribution of Leaves and Crowns to Water Shortage Coping Strategies
Source: Front Plant Sci. 2016 Dec 27;7:1958. doi: 10.3389/fpls.2016.01958 (PMC5187378; doi:10.3389/fpls.2016.01958)
Supplement: Supplementary file 5 [file Table_4.DOCX]

### Supplementary Table 4

### Genes whose expression is reduced in Tad relative to Amu in leaf

| ID^a^ | Tad x Amu Log2 FC^b^ | | Affymetrix annotation^c^ | AGI^d^ |
| --- | --- | --- | --- | --- |
|  | **crown** | **leaf** |  |  |
| Contig3501_at | -0.124 | -5.035 | BEST BLASTX NR: 01/29/03 Q00531 e-108 60 kDa jasmonate-induced protein pir\|\|S25092 jasmonate-induced protein - barley | AT5G01280.1 |
| EBem09_SQ006_L23_at | -0.89 | -2.134 | BEST BLASTX NR: 10/13/02 T03731 3e-05 abscisic acid-induced protein - rice emb\|CAA61981.1\| EFA27 for EF hand, abscisic acid, 27kD [Oryza sativa] | AT5G55240.1 |
| rbaal10h14_at | -0.636 | -2.569 | BEST BLASTX NR: 10/02/02 T03731 2e-53 abscisic acid-induced protein - rice emb\|CAA61981.1\| EFA27 for EF hand, abscisic acid, 27kD [Oryza sativa] | AT4G26740.1 |
| Contig3691_at | 0.624 | -2.276 | BEST BLASTX NR: 11/06/02 T04419 e-135 aleurone ribonuclease (EC 3.1.-.-) - barley (fragment) gb\|AAB58718.1\| aleurone ribonuclease [Hordeum vulgare] | AT2G02990.1 |
| Contig8739_at | -0.718 | -2.147 | BEST BLASTX NR: 11/08/02 AAG28786.1 2e-99 (AF308474) asparaginase [Hordeum vulgare] [Hordeum vulgare subsp. vulgare] | AT3G16150.1 |
| Contig8740_at | -0.365 | -2.125 | BEST BLASTX NR: 11/04/02 AAG28786.1 2e-71 (AF308474) asparaginase [Hordeum vulgare] [Hordeum vulgare subsp. vulgare] | AT3G16150.1 |
| Contig11160_at | 0.158 | -2.075 | BEST BLASTX NR: 11/08/02 AAM78094.1 2e-57 (AY125502) AT5g47240/MQL5_10 [Arabidopsis thaliana] gb\|AAN18132.1\| (BT000563) At5g47240/MQL5_10 [Arabidopsis thaliana] | AT5G47240.1 |
| Contig2087_s_at | 0.3 | -2.279 | BEST BLASTX NR: 11/06/02 P12940 2e-66 BOWMAN-BIRK TYPE TRYPSIN INHIBITOR pir\|\|TIBHB trypsin inhibitor (Bowman-Birk) - two-rowed barley | AT2G40070.2 |
| Contig15369_at | 0.18 | -2.375 | BEST BLASTX NR: 10/27/02 NP_568246.1 7e-41 bZIP protein HY5; protein id: At5g11260.1, supported by cDNA: gi_2244708, supported by cDNA: gi_2251084 [Arabidopsis | AT5G11260.1 |
| Contig8067_at | 0.352 | -3.35 | BEST BLASTX NR: 10/26/02 AAL25129.1 1e-94 cellulose synthase-like protein OsCslE1 [Oryza sativa] | AT1G55850.1 |
| Contig3112_at | -0.537 | -2.099 | BEST BLASTX NR: 10/13/02 T06810 2e-87 cold acclimation protein WCOR413 - wheat gb\|AAB18207.1\| cold acclimation protein WCOR413 [Triticum aestivum] | AT2G15970.1 |
| Contig14754_at | 0.212 | -3.135 | BEST BLASTX NR: 11/06/02 BAB92571.1 8e-43 (AP003380) contains EST AU070785~similar to Arabidopsis thaliana chromosome 3, At3g48360~unknown protein [Oryza sativa |  |
| Contig3900_at | 0.388 | -2.035 | BEST BLASTX NR: 11/06/02 T06207 e-137 cysteine proteinase (EC 3.4.22.-) - barley emb\|CAB09698.1\| cysteine proteinase [Hordeum vulgare subsp. vulgare] | AT5G45890.1 |
| Contig5434_at | -0.986 | -2.542 | BEST BLASTX NR: 10/02/02 BAB92670.1 2e-21 (AP003433) cysteine proteinase inhibitor Scb-like protein [Oryza sativa (japonica cultivar-group)] | AT2G31980.1 |
| Contig13712_at | 0.082 | -2.575 | BEST BLASTX NR: 11/07/02 BAA96757.1 8e-20 (AP002521) ESTs AU057825(S21823),AU057072(S21123) correspond to a region of the predicted gene.~Similar to Nicotiana |  |
| Contig3674_at | 0.144 | -2.042 | BEST BLASTX NR: 10/26/02 T04175 e-101 expansin - rice gb\|AAB81662.1\| expansin [Oryza sativa] | AT1G69530.2 |
| Contig3675_at | 1.061 | -2.888 | BEST BLASTX NR: 11/04/02 CAC06433.1 1e-92 (AJ276007) expansin [Festuca pratensis] | AT1G69530.2 |
| Contig4250_s_at | -0.613 | -2.328 | BEST BLASTX NR: 10/13/02 AAF25968.1 1e-71 F6N18.8 [Arabidopsis thaliana] | AT1G32700.1 |
| Contig2492_s_at | -0.56 | -3.069 | BEST BLASTX NR: 11/08/02 AAL92880.1 2e-42 fructosyltransferase [Lolium perenne] | AT1G62660.1 |
| rbah48h06_s_at | -0.425 | -3.306 | BEST BLASTX NR: 10/13/02 AAL92880.1 6e-84 fructosyltransferase [Lolium perenne] | AT1G62660.1 |
| Contig3156_s_at | 1.598 | -2.778 | BEST BLASTX NR: 11/04/02 T05956 7e-36 germin-like protein - barley emb\|CAA63659.1\| (X93171) oxalate oxidase-like protein or germin-like protein | AT5G39110.1 |
| Contig2768_s_at | -0.087 | -2.996 | BEST BLASTX NR: 11/08/02 T05721 e-110 germin-like protein 1 - barley emb\|CAA75907.1\| Germin-like protein 1 [Hordeum vulgare subsp. vulgare] | AT1G72610.1 |
| Contig3548_at | 0.761 | -3.903 | BEST BLASTX NR: 11/08/02 T01354 9e-51 herbicide safener binding protein 1 - maize gb\|AAC12715.1\| herbicide safener binding protein [Zea mays] | AT4G35160.1 |
| Contig1179_at | -1.281 | -2.546 | BEST BLASTX NR: 11/06/02 P05621 5e-44 Histone H2B.2 pir\|\|HSWT2B histone H2B.2 - wheat | AT5G22880.1 |
| HS18L03u_at | 0.551 | -2.019 | BEST BLASTX NR: 11/04/02 AAL01165.1 6e-24 (AC079843) Hypothetical protein [Oryza sativa] gb\|AAN11201.1\| (AC099734) Hypothetical protein [Oryza sativa (japonica |  |
| Contig422_at | -0.124 | -2.246 | BEST BLASTX NR: 11/08/02 AAB18209.1 e-132 chlorophyll a/b-binding protein WCAB precursor [Triticum aestivum] | AT2G34420.1 |
| Contig425_at | 0.331 | -2.43 | BEST BLASTX NR: 11/07/02 AAB18209.1 e-113 chlorophyll a/b-binding protein WCAB precursor [Triticum aestivum] | AT2G34420.1 |
| baak16l04_x_at | 0.205 | -3.039 | BEST BLASTX NR: 11/08/02 P04782 3e-37 Chlorophyll A-B binding protein 25, chloroplast precursor (LHCII type I CAB-25) (LHCP) | AT2G34420.1 |
| Contig11656_at | -0.657 | -2.411 | BEST BLASTX NR: 11/06/02 NP_181316.1 1e-06 (NM_129336) CHP-rich zinc finger protein, putative; protein id: At2g37800.1 [Arabidopsis thaliana] | AT2G37800.1 |
| Contig5433_at | -0.034 | -2.425 | BEST BLASTX NR: 11/08/02 P49249 e-102 IN2-2 PROTEIN | AT1G60680.1 |
| Contig2900_at | 1.793 | -2.299 | BEST BLASTX NR: 10/13/02 S58215 4e-90 jasmonate induced protein - barley emb\|CAA58110.1\| jasmonate induced protein [Hordeum vulgare subsp. vulgare] | AT3G51430.2 |
| Contig26204_at | -0.214 | -3.672 | BEST BLASTX NR: 11/07/02 AAD34319.1 2e-66 (AF105149) kaurene synthase [Zea mays] | AT1G79460.1 |
| Contig8538_at | 0.689 | -2.078 | BEST BLASTX NR: 11/08/02 Q99090 2e-35 Light-inducible protein CPRF-2 emb\|CAA41453.1\| (X58577) DNA-binding protein; bZIP type [Petroselinum crispum] | AT5G28770.2 |
| AF069331_s_at | -0.427 | -2.933 | BEST BLASTX NR: 11/06/02 AAC96100.2 2e-23 low temperature induced protein [Hordeum vulgare] |  |
| Contig6859_at | -0.172 | -2.728 | BEST BLASTX NR: 01/29/03 AAC96100.2 2e-23 low temperature induced protein [Hordeum vulgare] |  |
| Contig15753_at | -0.56 | -2.272 | BEST BLASTX NR: 11/04/02 AAB87721.1 6e-34 (U40489) maize gl1 homolog [Arabidopsis thaliana] | AT1G02205.2 |
| Contig14229_at | 0.398 | -2.335 | BEST BLASTX NR: 10/29/02 S52003 3e-99 major intrinsic protein - rice dbj\|BAA04257.1\| major intrinsic protein [Oryza sativa] | AT4G18910.1 |
| HM10M06r_at | 0.295 | -2.089 | BEST BLASTX NR: 10/26/02 NP_683476.1 5e-04 (NM_148635) MATE efflux family protein, putative; protein id: At1g66760.2 [Arabidopsis thaliana] | AT3G23560.1 |
| HS16E24u_s_at | -1.399 | -2.068 | BEST BLASTX NR: <none> | AT1G35910.1 |
| Contig2499_s_at | -1.393 | -5.078 | BEST BLASTX NR: <none> | AT1G62660.1 |
| HS06C19u_s_at | -1.346 | -2.504 | BEST BLASTX NR: <none> | AT2G31980.1 |
| Contig7003_at | -0.776 | -2.249 | BEST BLASTX NR: <none> | AT5G17210.2 |
| HS16K10u_at | -0.643 | -2.054 | BEST BLASTX NR: <none> |  |
| Contig23669_at | -0.632 | -2.263 | BEST BLASTX NR: <none> |  |
| HVSMEb0005B20r2_s_at | -0.32 | -3.341 | BEST BLASTX NR: <none> |  |
| Contig2496_at | -0.168 | -2.814 | BEST BLASTX NR: <none> |  |
| Contig9923_at | -0.07 | -3.714 | BEST BLASTX NR: <none> |  |
| Contig2632_s_at | -0.06 | -2.15 | BEST BLASTX NR: <none> | AT2G44710.1 |
| HO14H22S_s_at | -0.058 | -2.321 | BEST BLASTX NR: <none> | AT2G36630.1 |
| Contig2630_at | -0.022 | -2.361 | BEST BLASTX NR: <none> |  |
| rbaal21f05_s_at | -0.003 | -4.407 | BEST BLASTX NR: <none> | AT5G45890.1 |
| Contig12460_at | 0.018 | -2.169 | BEST BLASTX NR: <none> | AT4G31580.2 |
| Contig9922_s_at | 0.079 | -3.781 | BEST BLASTX NR: <none> |  |
| Contig7209_at | 0.097 | -2.56 | BEST BLASTX NR: <none> |  |
| Contig23882_at | 0.098 | -2.307 | BEST BLASTX NR: <none> |  |
| HS14L06r_at | 0.103 | -2.358 | BEST BLASTX NR: <none> |  |
| HY09M19u_s_at | 0.142 | -2.513 | BEST BLASTX NR: <none> | AT1G18100.1 |
| Contig11540_at | 0.359 | -2.305 | BEST BLASTX NR: <none> |  |
| Contig4544_s_at | 0.371 | -2.264 | BEST BLASTX NR: <none> | AT3G55646.1 |
| HS05F12u_s_at | 0.58 | -2.54 | BEST BLASTX NR: <none> | AT1G69530.2 |
| Contig3901_s_at | 0.751 | -3.027 | BEST BLASTX NR: <none> | AT5G45890.1 |
| Contig10680_at | 0.801 | -2.269 | BEST BLASTX NR: <none> |  |
| Contig7305_at | 0.18 | -2.413 | BEST BLASTX NR: 11/06/02 P47917 4e-46 O-METHYLTRANSFERASE ZRP4 (OMT) pir\|\|JQ2268 O-methyltransferase (EC 2.1.1.-) - maize | AT4G35160.1 |
| Contig18141_at | -1.762 | -2.399 | BEST BLASTX NR: 10/02/02 CAD39838.1 2e-68 OSJNBb0072N21.4 [Oryza sativa (japonica cultivar-group)] | AT1G19250.1 |
| Contig21141_at | 0.363 | -4.49 | BEST BLASTX NR: 10/13/02 CAD39838.1 1e-42 OSJNBb0072N21.4 [Oryza sativa (japonica cultivar-group)] | AT1G19250.1 |
| Contig6235_s_at | -0.736 | -2.301 | BEST BLASTX NR: 11/06/02 BAA89800.1 6e-06 (AB028185) OsNAC6 protein [Oryza sativa] gb\|AAK17067.1\| (AF254558) NAC6 [Oryza sativa] [Oryza sativa (japonica | AT1G01720.1 |
| Contig6233_at | -0.544 | -2.414 | BEST BLASTX NR: 11/07/02 BAA89800.1 2e-50 (AB028185) OsNAC6 protein [Oryza sativa] gb\|AAK17067.1\| (AF254558) NAC6 [Oryza sativa] [Oryza sativa (japonica | AT1G01720.1 |
| Contig6233_s_at | -0.214 | -2.031 | BEST BLASTX NR: 11/07/02 BAA89800.1 2e-50 (AB028185) OsNAC6 protein [Oryza sativa] gb\|AAK17067.1\| (AF254558) NAC6 [Oryza sativa] [Oryza sativa (japonica | AT1G01720.1 |
| Contig4545_s_at | -0.009 | -2.188 | BEST BLASTX NR: 11/04/02 BAB64285.1 2e-20 P0005H10.22 [Oryza sativa (japonica cultivar-group)] | AT3G55646.1 |
| Contig2113_at | 0.199 | -2.129 | BEST BLASTX NR: 11/06/02 AAC49819.1 2e-82 (AF014468) peroxidase [Oryza sativa] | AT5G05340.1 |
| HVSMEg0002E24r2_s_at | 0.417 | -3.49 | BEST BLASTX NR: 11/08/02 BAA33810.1 8e-04 phi-1 [Nicotiana tabacum] | AT4G08950.1 |
| Contig3241_at | -0.007 | -2.675 | BEST BLASTX NR: 01/29/03 T09164 5e-47 probable peroxidase (EC 1.11.1.7) (clone PC44) - spinach emb\|CAA71491.1\| peroxidase [Spinacia oleracea] | AT5G05340.1 |
| HVSMEm0015M15r2_s_at | -0.12 | -2.099 | BEST BLASTX NR: 11/04/02 T05968 3e-13 probable phenylalanine ammonia-lyase (EC 4.3.1.5) - barley (fragment) | AT2G37040.1 |
| Contig2490_at | -0.737 | -4.522 | BEST BLASTX NR: 01/29/03 T06338 e-131 probable vacuolar hydrolase (EC 3.2.1.-) - wheat (fragment) emb\|CAA12061.1\| beta-fructosidase [Triticum aestivum] | AT1G62660.1 |
| Contig9533_at | -0.054 | -2.534 | BEST BLASTX NR: 01/29/03 H96714 4e-55 protein F4N2.2 [imported] - Arabidopsis thaliana gb\|AAF27052.1\|AC008262_1 F4N2.2 [Arabidopsis thaliana] | AT1G69040.2 |
| Contig8058_at | -1.333 | -2.123 | BEST BLASTX NR: 11/04/02 Q00747 7e-07 PROTEIN LE25 pir\|\|S19253 gene le25 protein - tomato | AT5G06760.1 |
| Contig13201_at | -0.02 | -3.172 | BEST BLASTX NR: 11/04/02 NP_568919.1 2e-15 pseudo-response regulator - like; protein id: At5g60100.1, supported by cDNA: gi_10281007 [Arabidopsis thaliana] | AT5G60100.2 |
| Contig1616_at | 1.817 | -2.179 | BEST BLASTX NR: 11/06/02 AAK20055.1 2e-92 (AC025783) putative cellulase [Oryza sativa (japonica cultivar-group)] | AT3G44990.1 |
| Contig5196_at | -0.939 | -4.904 | BEST BLASTX NR: 11/06/02 BAB90447.1 e-111 putative CTP synthase [Oryza sativa (japonica cultivar-group)] | AT3G12670.1 |
| Contig11207_at | -0.154 | -2.696 | BEST BLASTX NR: 11/07/02 BAA99523.1 1e-42 putative cytochrome P450 [Oryza sativa (japonica cultivar-group)] | AT4G39490.1 |
| HV_CEa0008F11r2_at | 0.918 | -2.773 | BEST BLASTX NR: 11/04/02 BAC07345.1 1e-21 (AP003344) putative cytokinin oxidase [Oryza sativa (japonica cultivar-group)] | AT1G75450.1 |
| Contig23773_at | 0.746 | -4.695 | BEST BLASTX NR: 11/07/02 AAK53837.1 8e-42 (AC011806) Putative glutaredoxin [Oryza sativa] dbj\|BAB64731.1\| (AP003236) hypothetical protein [Oryza sativa (japonica | AT5G18600.1 |
| Contig15595_at | 0.061 | -2.6 | BEST BLASTX NR: 10/29/02 BAB68070.1 2e-45 (AP003560) putative heat shock transcription factor [Oryza sativa (japonica cultivar-group)] | AT3G24520.1 |
| Contig12563_s_at | 0.271 | -2.786 | BEST BLASTX NR: 11/07/02 AAL77133.1 6e-32 Putative chalcone synthase [Oryza sativa] | AT5G13930.1 |
| Contig16381_at | 0.096 | -2.126 | BEST BLASTX NR: 10/13/02 BAB17195.1 4e-50 (AP002855) putative isoflavone reductase [Oryza sativa (japonica cultivar-group)] | AT4G39230.1 |
| Contig14006_at | -0.671 | -2.054 | BEST BLASTX NR: 11/06/02 NP_196670.1 9e-06 (NM_121147) putative protein; protein id: At5g11090.1, supported by cDNA: 32958. [Arabidopsis thaliana] |  |
| Contig11328_at | -0.348 | -2.065 | BEST BLASTX NR: 11/06/02 NP_567108.1 5e-15 (NM_115970) putative protein; protein id: At3g61060.1, supported by cDNA: gi_14334587 [Arabidopsis thaliana] | AT3G61060.1 |
| Contig12484_at | 0.146 | -2.611 | BEST BLASTX NR: 11/07/02 NP_191084.1 4e-19 putative protein; protein id: At3g55240.1, supported by cDNA: gi_19310582 [Arabidopsis thaliana] | AT3G55240.1 |
| Contig18997_at | -0.496 | -3.359 | BEST BLASTX NR: 11/08/02 AAM22740.1 1e-33 (AC092388) putative serine/threonine protein kinase [Oryza sativa (japonica cultivar-group)] | AT2G05940.1 |
| Contig13848_at | -0.197 | -4.267 | BEST BLASTX NR: 11/04/02 BAB89883.1 9e-86 (AP003299) putative subtilase [Oryza sativa (japonica cultivar-group)] | AT5G11940.1 |
| Contig9702_at | 0.048 | -2.104 | BEST BLASTX NR: 10/13/02 AAL03950.1 e-102 (AY046898) relA/spoT-like protein RSH1 [Nicotiana tabacum] | AT3G14050.1 |
| Contig13847_s_at | 0.221 | -3.354 | BEST BLASTX NR: 11/08/02 T05928 1e-51 subtilisin-like proteinase (EC 3.4.21.-) - barley (fragment) emb\|CAA10987.1\| (AJ222782) subtilisin-like protease [Hordeum vulgare subsp. | AT5G11940.1 |
| Contig10552_at | -0.104 | -2.84 | BEST BLASTX NR: 11/08/02 NP_181201.2 4e-21 (NM_129218) unknown protein; protein id: At2g36630.1, supported by cDNA: gi_18700128 [Arabidopsis thaliana] | AT2G36630.1 |
| HT11N18r_s_at | -0.935 | -2.442 | BEST BLASTX NR: 10/26/02 T04351 1e-09 viviparous-14 protein - maize gb\|AAB62181.1\| (U95953) viviparous-14 [Zea mays] | AT1G78390.1 |
| Contig2007_s_at* | -2.677 | -2.563 | BEST BLASTX NR: 10/13/02 CAA69172.1 2e-63 17 kDa class I small heat shock protein [Hordeum vulgare subsp. vulgare] | AT5G59720.1 |
| Contig3685_at* | -3.732 | -3.316 | BEST BLASTX NR: 10/26/02 T06978 3e-77 ABA-induced plasma membrane protein PM 19 - wheat gb\|AAB38504.1\| ABA induced plasma membrane protein PM 19 [Triticum aestivum] | AT1G04560.1 |
| Contig3684_at* | -6.147 | -2.1 | BEST BLASTX NR: 11/06/02 T06978 5e-74 ABA-induced plasma membrane protein PM 19 - wheat gb\|AAB38504.1\| ABA induced plasma membrane protein PM 19 [Triticum aestivum] | AT1G04560.1 |
| Dhn10(Morex)_s_at* | -3.199 | -2.265 | BEST BLASTX NR: 11/07/02 AAF01698.1 1e-59 (AF181460) dehydrin; DHN10 [Hordeum vulgare] [Hordeum vulgare subsp. vulgare] | AT3G50970.1 |
| EBro08_SQ011_I03_at* | -2.267 | 2.152 | BEST BLASTX NR: 11/06/02 T05740 9e-26 heat shock protein 18 - barley emb\|CAA45862.1\| 18 Kd heat shock protein [Hordeum vulgare subsp. vulgare] | AT5G59720.1 |
| Contig3431_x_at* | -2.212 | -4.423 | BEST BLASTX NR: 11/06/02 P14897 7e-63 Low molecular mass early light-inducible protein HV90, chloroplast precursor (ELIP) | AT3G22840.1 |
| Contig8119_at* | -6.564 | -5.208 | BEST BLASTX NR: <none> |  |
| Contig7004_at* | -2.719 | -2.614 | BEST BLASTX NR: <none> | AT5G17210.2 |
| rbaal9h21_s_at* | -2.271 | -3.166 | BEST BLASTX NR: 10/02/02 Q40070 2e-33 Photosystem II 10 kDa polypeptide, chloroplast precursor pir\|\|T06173 photosystem II 10K protein precursor - barley | AT1G79040.1 |
| Contig11477_at* | -2.242 | -3.028 | BEST BLASTX NR: 11/08/02 Q40070 5e-39 Photosystem II 10 kDa polypeptide, chloroplast precursor pir\|\|T06173 photosystem II 10K protein precursor - barley | AT1G79040.1 |
| Contig26042_at* | -2.075 | -2.84 | BEST BLASTX NR: 11/08/02 CAD24007.1 .068 WIRE protein [Homo sapiens] |  |
| rbags15p13_s_at* | 2.941 | -3.605 | BEST BLASTX NR: 11/08/02 P32024 9e-30 23 KD JASMONATE-INDUCED PROTEIN pir\|\|S22514 jasmonate-induced protein 1 - barley |  |
| Contig3775_s_at* | 3.669 | -3.234 | BEST BLASTX NR: 11/06/02 BAB16431.1 8e-15 P-rich protein Nt-SubC29 [Nicotiana tabacum] |  |

* Significant difference in both crown and leaf

^a^ Affymetrix 22 K Barley1 GeneChip Genome Array probe ID

^b^ Log2 transformed expression difference of Tad against Amu in crown/leaf

^c^ Microarray manufacturer (Affymetrix) annotation of individual IDs

^d^*Arabidopsis* locus identifier corresponding to individual IDs
