# Supplementary material for: Global Scale Transcriptional Profiling of Two Contrasting Barley Genotypes Exposed to Moderate Drought Conditions: Contribution of Leaves and Crowns to Water Shortage Coping Strategies
Source: Front Plant Sci. 2016 Dec 27;7:1958. doi: 10.3389/fpls.2016.01958 (PMC5187378; doi:10.3389/fpls.2016.01958)
Supplement: Supplementary file 6 [file Table_5.DOCX]

### Supplementary Table 5

### Genes whose expression is enhanced in Tad relative to Amu in crown

| ID^a^ | Tad x Amu Log2 FC^b^ | | Affymetrix annotation^c^ | AGI^d^ |
| --- | --- | --- | --- | --- |
|  | **crown** | **leaf** |  |  |
| Contig2329_x_at | 2.027 | 1.562 | BEST BLASTX NR: 11/06/02 NP_177794.1 4e-84 12-oxophytodienoate reductase (OPR1); protein id: At1g76680.1, supported by cDNA: 38527., supported by cDNA: | AT1G76680.1 |
| Contig1679_s_at | 2.571 | -1.421 | BEST BLASTX NR: 10/27/02 P32024 8e-93 23 KD JASMONATE-INDUCED PROTEIN pir\|\|S22514 jasmonate-induced protein 1 - barley |  |
| rbags15p13_s_at* | 2.941 | -3.605 | BEST BLASTX NR: 11/08/02 P32024 9e-30 23 KD JASMONATE-INDUCED PROTEIN pir\|\|S22514 jasmonate-induced protein 1 - barley |  |
| Contig11252_at | 2.122 | -0.739 | BEST BLASTX NR: 10/02/02 BAB60849.1 2e-25 (AB062746) BURP domain-containing protein [Bruguiera gymnorrhiza] | AT5G25610.1 |
| Contig5312_at | 2.33 | -0.024 | BEST BLASTX NR: 11/04/02 AAD10254.1 e-108 (AF033539) caffeic acid O-methyltransferase; LPOMT2 [Lolium perenne] | AT5G54160.1 |
| Contig11421_at | 3.135 | -0.058 | BEST BLASTX NR: 11/06/02 NP_405235.1 9e-26 (NC_003143) cold shock protein [Yersinia pestis] emb\|CAC90477.1\| (AJ414149) cold shock protein [Yersinia pestis] |  |
| Contig14008_at | 2.158 | 0.898 | BEST BLASTX NR: 11/06/02 BAB19383.1 8e-10 (AP002542) contains EST AU076248(S20152)~unknown protein [Oryza sativa (japonica cultivar-group)] |  |
| HB25K10r_at | 2.979 | 0.326 | BEST BLASTX NR: 10/27/02 S61414 2e-06 DNA-binding protein ABF2 - wild oat emb\|CAA88331.1\| (Z48431) DNA-binding protein [Avena fatua] | AT1G80840.1 |
| HS17D15r_s_at | 2.444 | -0.315 | BEST BLASTX NR: 10/26/02 BAA83560.1 2e-23 (AP000399) EST AU077941(C12908) corresponds to a region of the predicted gene.~Similar to OsENOD93a gene for early | AT5G25940.1 |
| HB27C21r_s_at | 2.089 | 0.101 | BEST BLASTX NR: 10/31/02 BAA83566.1 1e-33 ESTs C98280(C1391),D15843(C1391) correspond to a region of the predicted gene.~Similar to OsENOD93a gene for early | AT5G25940.1 |
| Contig10558_at | 2.298 | -0.042 | BEST BLASTX NR: 10/27/02 NP_563851.1 2e-42 (NM_100847) expressed protein; protein id: At1g09750.1, supported by cDNA: 6295. [Arabidopsis thaliana] | At1g09750.1 |
| Contig141_at | 2.29 | -0.283 | BEST BLASTX NR: 11/04/02 P02300 2e-64 Histone H3 pir\|\|S04520 histone H3 (clone pH3c-1) - alfalfa | AT5G65360.1 |
| Contig159_at | 2.292 | -0.374 | BEST BLASTX NR: 10/13/02 HSWT41 4e-47 histone H4 (TH091) - wheat gb\|AAA34292.1\| histone H4 | AT5G59970.1 |
| Contig7887_at | 3.682 | 0.039 | BEST BLASTX NR: 11/06/02 T04375 e-168 jacalin homolog - barley gb\|AAA87042.1\| (U43497) putative 32.7 kDa jasmonate-induced protein [Hordeum | AT1G19715.3 |
| Contig7886_at | 4.885 | 0.178 | BEST BLASTX NR: 10/29/02 T04374 e-107 jakalin homolog - barley gb\|AAA87041.1\| (U43496) putative 32.6 kDa jasmonate-induced protein [Hordeum | AT1G19715.3 |
| Contig2899_s_at | 2.1 | 0.925 | BEST BLASTX NR: 10/27/02 S58215 6e-93 jasmonate induced protein - barley emb\|CAA58110.1\| jasmonate induced protein [Hordeum vulgare subsp. vulgare] |  |
| Contig9234_at | 2.195 | 1.756 | BEST BLASTX NR: <none> |  |
| HK05P14r_s_at | 2.489 | 0.679 | BEST BLASTX NR: <none> | AT4G27520.1 |
| Contig14219_at | 2.654 | 0.346 | BEST BLASTX NR: <none> | AT2G28671.1 |
| Contig12634_at | 2.095 | 0.221 | BEST BLASTX NR: <none> |  |
| HS06A14u_s_at | 2.273 | 0.12 | BEST BLASTX NR: <none> | AT5G25940.1 |
| HS07M18u_s_at | 2.111 | 0.062 | BEST BLASTX NR: <none> |  |
| HVSMEa0002K15r2_s_at | 2.902 | -0.002 | BEST BLASTX NR: <none> | AT4G34215.2 |
| Contig17920_at | 2.071 | -0.048 | BEST BLASTX NR: <none> |  |
| HZ49G19r_at | 3.072 | -0.156 | BEST BLASTX NR: <none> | AT5G65360.1 |
| rbags24b14_at | 2.229 | -0.298 | BEST BLASTX NR: <none> |  |
| Contig13248_at | 2.986 | -0.485 | BEST BLASTX NR: 11/06/02 CAD40043.1 1e-82 (AL606590) OSJNBa0052O21.28 [Oryza sativa (japonica cultivar-group)] | AT1G05680.1 |
| Contig25762_at | 2.502 | 1.96 | BEST BLASTX NR: 11/06/02 BAC16424.1 4e-22 P0045F02.11 [Oryza sativa (japonica cultivar-group)] | AT5G48485.1 |
| rbah13p07_s_at* | 2.605 | 2.332 | BEST BLASTX NR: 10/02/02 AAM76682.1 2e-24 (AF387866) peroxidase [Triticum aestivum] | AT5G05340.1 |
| Contig2112_at* | 2.374 | 2.257 | BEST BLASTX NR: 11/07/02 S61406 e-103 peroxidase (EC 1.11.1.7) 2 precursor - wheat emb\|CAA59485.1\| (X85228) peroxidase [Triticum aestivum] | AT5G05340.1 |
| Contig3775_s_at* | 3.669 | -3.234 | BEST BLASTX NR: 11/06/02 BAB16431.1 8e-15 P-rich protein Nt-SubC29 [Nicotiana tabacum] |  |
| HU12P09u_s_at | 2.055 | 0.475 | BEST BLASTX NR: 11/04/02 BAA90877.1 .010 (AB031227) PsAD1 [Pisum sativum] | AT1G16610.3 |
| Contig4020_at | 2.328 | -0.208 | BEST BLASTX NR: 10/29/02 BAB17110.1 3e-85 (AP002866) putative acetyl transferase [Oryza sativa (japonica cultivar-group)] | AT3G62160.1 |
| Contig7990_at | 2.639 | 0.304 | BEST BLASTX NR: 11/06/02 AAK38502.1 4e-36 putative basic protein [Oryza sativa] | AT2G02850.1 |
| Contig9564_at | 3.557 | 0.108 | BEST BLASTX NR: 10/29/02 AAG13488.1 8e-26 (AC026758) putative lipid transfer protein [Oryza sativa (japonica cultivar-group)] | AT4G12500.1 |
| Contig112_at | 2.307 | 0.118 | BEST BLASTX NR: 11/08/02 NP_197783.1 3e-33 (NM_122300) putative protein; protein id: At5g23950.1 [Arabidopsis thaliana] | AT4G22505.1 |
| Contig3533_at | 2.089 | 0.22 | BEST BLASTX NR: 10/13/02 S24263 2e-14 seed storage protein, 35K isoform AmA1 - prince's feather pir\|\|A47185 storage protein isoform AmA1, 35K - prince's feather |  |
| Contig9197_at | 2.053 | -0.296 | BEST BLASTX NR: 10/27/02 Q00497 4e-65 Shikimate kinase, chloroplast precursor pir\|\|S21584 shikimate kinase (EC 2.7.1.71) precursor - tomato | AT2G21940.5 |
| Contig1570_s_at | 3.509 | 0.228 | BEST BLASTX NR: 11/08/02 AAB21531.1 1e-76 thionin [Hordeum vulgare=barley, ssp. vulgare, leaf, cv. Carina, Peptide, 137 aa] | AT1G66100.1 |
| Contig7007_s_at | 2.964 | -0.166 | BEST BLASTX NR: 10/02/02 BAB93111.1 6e-10 (AB072337) thionin Osthi1 [Oryza sativa (japonica cultivar-group)] | AT2G15010.1 |
| Contig13867_s_at | 4.157 | 1.295 | BEST BLASTX NR: 11/08/02 BAA94219.1 5e-61 unnamed protein product [Oryza sativa (japonica cultivar-group)] | AT1G28600.1 |
| Contig13867_at | 4.391 | 0.461 | BEST BLASTX NR: 11/08/02 BAA94219.1 5e-61 unnamed protein product [Oryza sativa (japonica cultivar-group)] | AT1G28600.1 |

* Significant difference in both crown and leaf

^a^ Affymetrix 22 K Barley1 GeneChip Genome Array probe ID

^b^ Log2 transformed expression difference of Tad against Amu in crown/leaf

^c^ Microarray manufacturer (Affymetrix) annotation of individual IDs

^d^*Arabidopsis* locus identifier corresponding to individual IDs
